# Corpus luteum presence in the bovine ovary increase intrafollicular progesterone concentration: consequences in follicular cells gene expression and follicular fluid small extracellular vesicles miRNA contents

**DOI:** 10.1186/s13048-024-01387-3

**Published:** 2024-03-18

**Authors:** Paola Maria da Silva Rosa, Alessandra Bridi, Giuliana de Ávila Ferronato, Cibele Maria Prado, Natália Marins Bastos, Juliano Rodrigues Sangalli, Flávio Vieira Meirelles, Felipe Perecin, Juliano Coelho da Silveira

**Affiliations:** Department of Veterinary Medicine, College of Animal Science and Food Engineering, University of São Paulo, Av. Duque de Caxias Norte, 225, Pirassununga, São Paulo 13635-900 Brazil

**Keywords:** Sex-steroids, MicroRNAs, Granulosa cells, Cumulus cells

## Abstract

**Background:**

It is well described that circulating progesterone (P4) plays a key role in several reproductive events such as oocyte maturation. However, during diestrus, when circulating P4 is at the highest concentrations, little is known about its local impact on the follicular cells such as intrafollicular P4 concentration due to corpus luteum (CL) presence within the same ovary. Based on that, our hypothesis is that the CL presence in the ovary during diestrus alters intrafollicular P4 concentrations, oocyte competence acquisition, follicular cells gene expression, and small extracellular vesicles (sEVs) miRNAs contents.

**Results:**

P4 hormonal analysis revealed that ipsilateral to the CL follicular fluid (iFF) presented higher P4 concentration compared to contralateral follicular fluid (cFF). Furthermore, oocyte maturation and miRNA biogenesis pathways transcripts (*ADAMTS-1* and *AGO2*, respectively) were increased in cumulus and granulosa cells of iFF, respectively. Nevertheless, a RT-PCR screening of 382 miRNAs showed that three miRNAs were upregulated and two exclusively expressed in sEVs from iFF and are predicted to regulate cell communication pathways. Similarly, seven miRNAs were higher and two exclusively expressed from cFF sEVs and are predicted to modulate proliferation signaling pathways.

**Conclusion:**

In conclusion, intrafollicular P4 concentration is influenced by the presence of the CL and modulates biological processes related to follicular cell development and oocyte competence, which may influence the oocyte quality. Altogether, these results are crucial to improve our knowledge about the follicular microenvironment involved in oocyte competence acquisition.

**Supplementary Information:**

The online version contains supplementary material available at 10.1186/s13048-024-01387-3.

## Introduction

Follicular development requires a synchronized regulation between cellular communication and endocrine factors, to generate a viable oocyte [1]. The antral follicle contains follicular fluid (FF), which is an ultrafiltrate from blood plasma and secretions from various follicular cells (oocyte, cumulus, granulosa, and theca cells) [2]. Progesterone (P4) is synthesized in bovine ovaries, by follicular and luteal cells at varying concentrations [3]. Following ovulation, the corpus luteum (CL) becomes the primary source of P4 which can traverse the basal lamina entering the extracellular environment [4]. The synthesis of P4 gradually intensifies with the development of follicles, primarily driven by CL production [4, 5]. This synthesis reaches its peak and sustains secretion during the late diestrus phase [4, 5]. In small ruminant species, it was demonstrated that the CL proximity increases uterine P4 concentrations, creating a P4 concentration gradient along the ipsilateral reproductive tract [6]. Additionally, the CL presence also impacts FF composition such as progesterone (P4), which during follicular development can modulate cell-to-cell communication mediated by small extracellular vesicles (sEVs) in the FF [7]. Although, high circulating concentrations of P4 present in pregnant animals during a follicular emergence can improves pregnancy rates [8], the CL presence concurrently decreased cumulus cells expansion, blastocysts and pregnancy rate in non-pregnant animals [8–11]. Thus, a local P4 modulation of oocyte developmental competence mediated by intercellular communication through sEVs contents are still unknown.

The effects of the different intrafollicular P4 concentrations on cells and cell-to-cell communication within the ovarian follicle are still poorly understood. The oocyte development is triggered by the bidirectional cellular communication between the oocyte and its surrounding somatic cells [12]. This communication can take place through various mechanisms [13], such as paracrine signals mediated by steroids, proteins and metabolites secreted by both cell types [14]. Moreover, the release of sEVs by follicular cells during folliculogenesis also plays a crucial role facilitating communication among follicular cells [15]. sEVs are lipid bilayer membranes with a diameter ranging from 30 to 200 nm, secreted by the fusion of multivesicular bodies with the plasma membrane leading to the release to the extracellular environment [16]. Bioactive molecules, such as miRNAs, are carried by sEVs over large distances from secretory to target cells [17]. During folliculogenesis, smaller follicles (3-5 mm) exhibit higher concentrations of sEVs, with a progressive decrease observed as ovarian follicle development advances [18]. This implies a specific regulation of sEVs synthesis, secretion, and content loading during the follicular development. In addition to secretion, studies have shown that FF sEVs can be taken up by cumulus cells during in vitro maturation [19], and that it can improve cumulus cell expansion and increase the expression of cumulus expansion related-genes in a follicle stage dependent-manner [20]. These findings underscore the crucial role of FF sEVs in mediating communication among various types of follicular cells.

The sEVs effects are mainly mediated by their bioactive contents. Within the FF, a substantial portion of the detected miRNAs in the extracellular milieu are encapsulated within sEVs [21]. MiRNAs are short non-coding RNAs capable of regulating biological processes through a post-transcriptional silencing mechanism involving mRNA processing [22]. Previous data from our group demonstrated distinct miRNA profile in FF sEVs from follicles in different stages of the estrous cycle (exposed to high or low P4 concentration) [7]. Also, sEVs obtained from these FF with different P4 concentrations were predicted to modulate cumulus cell transcriptome after supplementation during *in vitro* maturation [7]. These findings suggest that sex steroid hormones, such as P4, may influence the contents of FF sEVs. However, the impact of elevated P4 concentrations, which occur during the same stage of the ovarian cycle due to the presence of the corpus luteum (CL), on intercellular communication within the follicular fluid via sEVs, remains poorly understood. Based on that, the hypothesis of our current study is that the presence of the corpus luteum (CL) leads to elevated intrafollicular progesterone (P4) concentrations in follicles on the same side as the CL, resulting in distinct modulation of transcripts in cumulus and granulosa cells, as well as distinct changes in the miRNA contents of FF sEVs when compared to contralateral follicles.

## Material and methods

Unless otherwise stated, all chemicals were purchased from Sigma‐Aldrich/Merck Chemical Company.

### Experimental design

To study the intrafollicular P4 effects on oocyte developmental competence, follicular cells gene expression and sEVs miRNA profile during the same estrous cycle stage (late diestrus – 11 to 16 days), slaughterhouse ovaries were collected in pairs (from the same cow) based on CL morphology (the CL exhibits a tan or orange color, without any discernible rupture point), as previously described [23] (Supplementary Figure S1). To obtain groups with different intrafollicular P4 concentrations based on CL presence, follicles measuring 3-6mm from ovaries either ipsilateral (Ipsi) or contralateral (Contra) to the CL were individually aspirated. This procedure was repeated in six biological replicates, each consisting of two to four pairs of ovaries, to obtain follicular fluid (iFF and cFF, respectively) and the P4 levels were evaluated in each group. A total of 254 COCs from Ipsi and 215 COCs from Contra follicles were morphologically evaluated and used for *in vitro* embryo production. Gene expression analysis (six biological replicates from each group) was evaluated in cumulus cells from five immature COCs per replicate and two pools of granulosa cell sheets (five sheets/pool) per replicate. Additionally, FF sEVs from each group were isolated and characterized by nanotracking analysis (NTA). Additional samples of sEVs were used to western blot, and transmission electron microscopy. A panel of 382 bovine miRNAs was analyzed in FF sEVs from six biological replicates (the same samples used for NTA analysis).

### Collection of ovarian follicular fluid

Pairs of ovaries were collected from a slaughterhouse near to Pirassununga region in the state of São Paulo and transported to the laboratory in saline solution within 3 h. Only pairs of ovaries that presented CL with morphological characteristics related to the late diestrus stage were used. Briefly, we used pairs of ovaries with the CL color ranging from orange to yellow in the external parenchyma with absence of large follicles (>8 mm). In this stage, cows present high and stable systemic progesterone concentration [23]. The follicular fluid from both groups (iFF and cFF) were aspirated with an 18G needle and 10 mL syringe and processed by differential centrifugation before freezing in freezer at -80°C until further use.

### Hormonal analysis, and groups formation

To characterize the different P4 intrafollicular environments and similar follicular health, P4 and E2 concentration from iFF and cFF were measured using the chemiluminescence assay (ADVIA Centaur-Siemens, Henkestr, Erlangen, Germany). Intraassay coefficients variations of P4 and E2 in follicular fluid were, 9.77 ng/mL and 11.06 ng/mL, respectively.

### Oocyte, cumulus and granulosa cells collection

The follicular aspirate from Ipsi and Contra follicles was deposited in a 100 mm petri dish, and pools of granulosa cell sheets and COCs were recovered with the aid of a stereoscope. COCs were initially washed and selected in TCM199 medium (GIBCO), buffered with HEPES (20 mM) supplemented with BSA (1%), sodium pyruvate (0.2 mM), and gentamycin (50 µg/mL). Finally, COCs were morphologically classified as previously described [24]. Briefly, the COCs were classified into grade 1 (G1), grade 2 (G2), grade 3 (G3), and non-viable oocytes (G4, and denuded). For the cumulus cells collection, ~20% of the cumulus cells layers of five immature G1 COCs per replicate were individually dissected with a 30G needle. The cumulus and granulosa cells were stored freezer at 80°C until further use.

### In vitro embryo production

Groups of COCs (n~25) were distributed into 100 µL of IVM medium drops composed of TCM199 buffered with 22 ug/mL of sodium bicarbonate supplemented with 0.4% of BSA, 0.5 μg/mL of follicle stimulating hormone, 5 UI/mL of human chorionic gonadotropin, 0.2 mM of sodium pyruvate and 50 μg/mL of gentamicin sulfate, under mineral oil. COCs were incubated at 38.5ºC, 5% CO_2_ in atmospheric air, and controlled humidity for 22-24 hours. After maturation, *in vitro* fertilization (IVF) was performed in IVF medium (TALP-IVF supplemented with 6mg/mL of BSA, 22 ug/mL of sodium pyruvate, 50 μg/mL of gentamicin, 5.5 UI/mL of heparin, 2mM of penicillamine, 1mM of hypotaurine and 245uM of epinephrine). COCs were distributed (n~25) in 100 µL of IVF drops, under mineral oil. Frozen semen was thawed at 36ºC and centrifuged in discontinuous Percoll (45-90%) at 3600 x g for 7 min. The supernatant was discarded, the pellet resuspended in IVF medium and, centrifuged at 520 x g for 5 min. Finally, the semen pellet was adjusted to a concentration of 5x10^3^ sperm per oocyte and was distributed among drops of IVF medium within COCs. The COCs were co-incubated for fertilization at 38.5ºC, 5% CO_2_ in atmospheric air, and controlled humidity for 18 h. The same semen batches were used in all experiments.

After fertilization, the presumptive zygotes were partially denuded by vigorous pipetting into the IVF drops and distributed (n~20) in drops (100 µL) of *in vitro* culture (IVC) medium (SOFaaci supplemented with 8mg/mL of BSA, 22 ug/mL of sodium pyruvate, and 50 μg/mL of gentamicin), under mineral oil and incubated at 38.5ºC, 5% CO_2_, 5% O_2_ and 90% N_2_ and controlled humidity. Next, 168 hours after IVF, the blastocyst rate was evaluated.

### Isolation of small extracellular vesicles from follicular fluid

The sEVs were isolated as previously described [25]. Briefly, 200 µL of iFF or cFF were centrifuged at 300 x g for 10 min, 2,000 x g for 10 min, and at 16,500 x g for 30 min, to remove live cells, cellular debris, and large vesicles, respectively. The remaining supernatant was placed in freezer at −80°C for further analysis. Upon use, supernatant was filtered through a 0.2 µm filter (PES membrane; Corning) and ultracentrifuged (Optima XE-90 Ultracentrifuge; rotor 70 Ti; Beckman Coulter) at 119.700 x g for 70 min twice to obtain an enriched pellet of sEVs. All centrifugation steps were performed at 4°C. The pellet was resuspended in 50 µL of phosphate-buffered saline (1× PBS Ca^2+^/Mg^2+^-free; 137 mM NaCl, 2.7 mM KCl, 10 mM Na_2_HPO_4_, 2 mM KH_2_PO_4_) and used for further analysis.

### Characterization of small extracellular vesicles

The characterization of sEVs isolation is a fundamental step to demonstrate the efficiency of the isolation protocol and to determine the presence of a sEVs enriched solution. The FF sEVs were characterized by particle size and concentration using nanoparticle tracking analysis (NTA), evaluation of specific and absent proteins was performed by western blotting analysis, and morphology by transmission electron microscopy (TEM).

#### Nanoparticle tracking analysis

Small extracellular vesicles pellet of iFF or cFF follicles of each replicate was resuspended in 50 µL of 1× PBS Ca^2+^/Mg^2+^-free. The concentration was adjusted for each sample and was measured using Nanosight (NS300; NTA 3.1 Build 3.1.45, Malvern, UK). The FF sEVs analysis by Nanosight was based on five videos of 30 seconds each. After recording these images, the equipment's software calculates size and concentration. Analyses were performed at 38.5°C, at the camera level 12, and threshold of 5 for all samples.

#### Western blot analysis

To conduct western blot analysis 500 µL of FF were used to obtain an enriched pellet of sEVs. The total protein (50 µg) from FF sEVs and follicular cells were isolated using RIPA (radioimmunoprecipitation assay) buffer and proteinase inhibitor cocktail and mixed with 4× Laemmli and beta-mercaptoethanol (Bio-rad, Hercules, CA). For protein denaturation, samples were incubated at 95°C for 5 min and loaded onto an SDS-PAGE 10% polyacrylamide gel. The gel run was performed at 100 V for 2 hours, and the protein samples were transferred onto a PVDF (Polyvinylidene Difluoride) membrane (1704156; Bio-RAD) at 80 V for 2 hours in a wet transfer apparatus. The protein membrane was washed three times in 1× Tris-buffered saline with Tween-20 (TBST) and incubated in a blocking buffer (5% of Non-Fat Dry Milk in TBST) at room temperature for 1 hour. The membrane was incubated overnight with a primary antibody at 4 °C. The proteins Alix (ALG-2 interacting protein X) and Tomm20 (translocase of outer mitochondrial membrane 20) were evaluated using a rabbit antibody against a peptide corresponding to an internal region of human Alix/ PDCD6IP (1:3000; SAB4200476; Sigma–Aldrich Chemical Company, St. Louis, MO) and a mouse monoclonal antibody raised against amino acids 1-145 of Tomm20 of human origin (1:6000; SC- 17764; Santa Cruz, CA), respectively. After incubation, the membrane was washed three times using 1× TBST for 5 min each and then incubated with secondary antirabbit (1:4000; A0545; Sigma–Aldrich Chemical Company, St. Louis, MO), and antimouse (1:4000; #7076S; Cell Signaling Technology, Danvers, Massachusetts, USA) antibody conjugates in horseradish peroxidase for 1 h at room temperature. Finally, the membrane was washed three times using 1× TBST and exposed to a detection solution (170–5060; Clarity Western ECL). The images were obtained using the ChemiDoc MP Image System (Bio-Rad, Hercules, CA).

#### Transmission electron microscopy (TEM)

An enriched pellet of sEVs was obtained from 100 µL of FF and used for the TEM analysis. The pellet (40 µL) was diluted in 400 µL of fixative solution (0.1 M cacodylate; 2.5% glutaraldehyde and 4% paraformaldehyde at pH 7.2–7.4) and incubated for 2 h at room temperature. To obtain sEVs fixed pellet, the solution was diluted in 2 mL of 1× PBS Ca^2+^/Mg^2+^-free and ultracentrifuged at 119.700 x g for 70 min, at 4°C. Next, sEVs pellets were diluted in 30 μL of milli-Q water and placed in a pioloform‐coated copper grid for approximately 60 min at room temperature. After completely dried, 2% of uranyl acetate was added to the grid for 90 s, the excess was removed with filter paper and then analyzed by TEM (FEI Tecnai 20; LAB6 emission; 200 kV).

### Total RNA extraction

Total RNA extraction of cumulus and granulosa cells as well as FF sEVs were performed using TRIzol® (Invitrogen) reagent according to the manufacturer’s instruction. The aqueous phase was recovered and added 1.33 µL GlycoBlue® (15 μg/ml; Thermo Fisher Scientific) an RNA co-precipitant to increase the RNA yield. The total RNA was treated with DNase I (Invitrogen, Carlsbad, CA), according to the manufacturer’s instructions to avoid genomic DNA contamination. RNA quality and concentration were analyzed using spectrometry (NanoDrop OneC; Thermo Fisher Scientific, Frederick, Maryland, USA). RNA quality was considered good when 260/280 was >1.7 in the nanodrop analysis.

### Reverse transcription, and real-time PCR

For mRNA RT-PCR, the reverse transcription reaction was performed using High-Capacity cDNA Reverse Transcription Kit (Thermo Fisher Scientific), according to the manufacturer’s protocol. For cDNA conversion, we used ~ 10 ng of the total RNA for each gene target of interest. For relative gene expression analysis, we selected genes involved in ovarian steroidogenesis, oocyte maturation, and miRNA biosynthesis pathways (Supplementary Table S1). In this analysis GoTaq qPCR Master Mix (Promega) was used according to the manufacturer’s instructions, with the following PCR cycle conditions: 95°C for 10min, 45 cycles of 95°C for 15 s, and 60°C for 60 s, followed by the melting curve.

Regarding, miRNA RT-PCR of FF sEVs, cDNA synthesis was performed using 200 ng of total RNA and with MystiCq® MicroRNA® Quantitation System (Sigma). For the relative miRNA expression analysis specific forward primers (Supplementary Table S2) were used as previously [7], the conditions were according to manufacturer’s instructions, with the following PCR cycle conditions: 95°C for 5 min, 45 cycles of 95°C for 10 s, 60°C for 30 s, and 70° for 30 s, followed by the melting curve. The levels of 383 miRNAs were evaluated in the six biological replicates per group. MiRNAs were considered exclusive when present at least in four biological samples of one group and at most in one sample of another group, or when it was present at least in three biological samples of one group and in none sample of another group.

For mRNA analysis, the amplification of single cDNA products was confirmed by the presence of a single melting peak, and for miRNA analysis, the amplification of cDNA products was confirmed by the presence of one or two melting peaks. Ct values smaller than 37 were used for both analyses. For mRNA RT-PCR, amplifications were normalized using the geometric mean of the Ct values of two endogenous genes (*PPIA* and *YWHAZ* for cumulus cells and PPIA and for granulosa cells), and for miRNA RT-PCR amplifications were normalized using the geometric mean of the Ct values of three miRNAs (miR-99b, RNU43 snoRNA and Hm/Ms/Rt U1 snRNA). All analyses were performed using 384 well plates in the QuantStudio 6 Flex (Applied Biosystems), the relative expression values were calculated using the ΔCt method, and the normalized data were transformed by 2^−**Δ**Ct^ for a graphical representation of the relative transcript levels. MicroRNA bioinformatic analyses were performed using Mirwalk software version 3.0. For predicted biological pathways identification we used exclusive and upregulated miRNAs of each group.

#### Statistical and bioinformatics analysis

The data were tested for outliers’ presence using the ROUT method and the normality for the Shapiro-Wilk test. COCs recovered and blastocyst rates were compared by Fisher test. Data of FF P4 and E2 concentration, relative expression of mRNAs in cumulus and granulosa cells, particle size, the concentration of FF sEVs, and relative expression of miRNAs were performed using Student t-test in the JMP 14 (SAS Institute Inc.) software, with a significance level of 5%.

## Results

### P4 concentration is higher in FF from ovaries ipsilateral to the CL

**Fig. 1:**
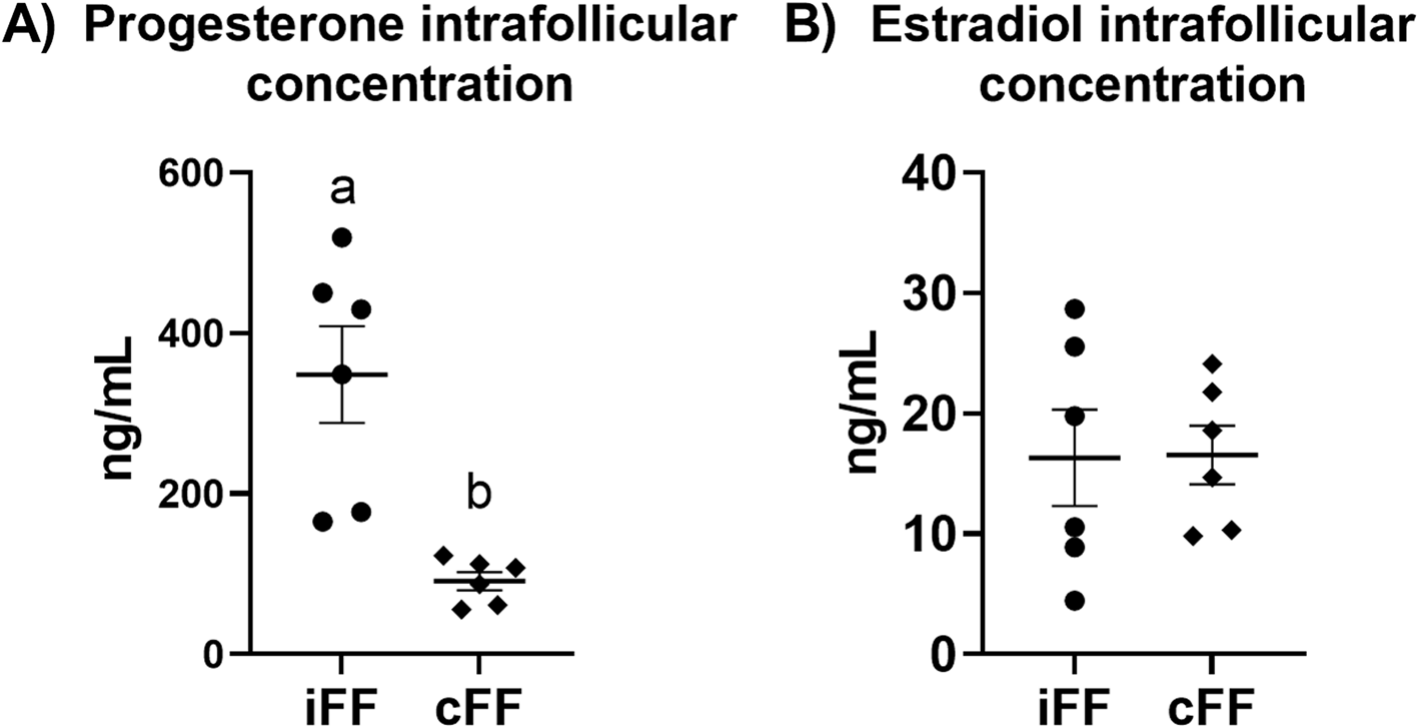
Progesterone and estradiol concentration in follicular fluid ipsilateral and contralateral to the corpus luteum ovaries. (**A**) Progesterone concentration (ng/mL) in iFF and cFF (*n*=6). (**B**) Estradiol concentration (ng/mL) in iFF and cFF (*n*=6). Points represent the value of each replicate. The black line between the error bars represents the group means and the error bars represent the standard error of the mean. Different letters indicate statistical differences (*p*<0.05)

To demonstrate high P4 concentration in iFF compared to cFF, the FF was collected from ovaries ipsilateral or contralateral to the CL at the diestrus cycle stage. The iFF had a higher P4 concentration (348.38 ± 61.32 ng/mL) when compared to cFF (91.06 ± 11.33 ng/mL; p=0.007) at the same estrous cycle stage (Fig 1A). Furthermore, E2 concentration was similar between iFF (16.31 ± 3.99 ng/mL) and cFF (16.55 ± 2.42 ng/mL) (Fig 1B). Altogether, these results demonstrate that follicular components are exposed to different P4 concentrations, i.e., follicular cells are exposed to high (iFF) and low P4 (cFF) concentrations, depending on CL presence or absence in the ovary.

### Oocyte morphological quality and blastocyst rates are the same regardless of CL presence in the ovary

**Fig. 2:**
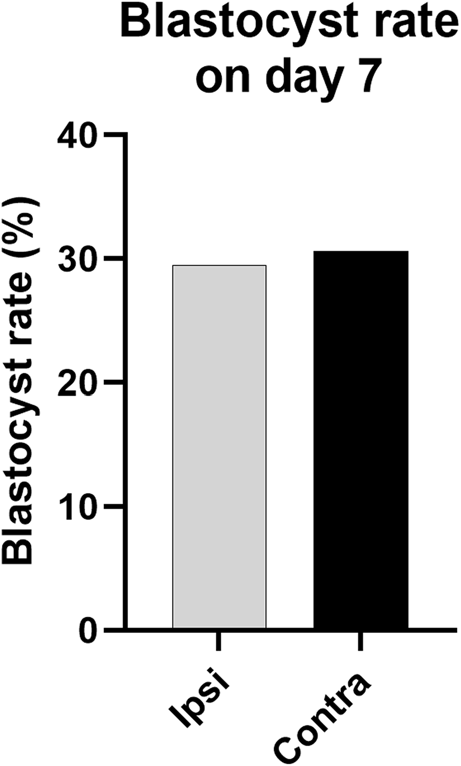
Blastocyst rate of oocytes from follicle Ipsi and contra to the corpus luteum. The blastocyst rate was calculated based on the number of blastocysts on day 7 of development per number of presumptive zygotes placed in in vitro culture from follicles Ipsi (*n*=54/183) and Contra (*n*=49/160) to the CL. We performed a total of 6 biological replicates

To analyze the effects of the CL presence or absence in the ovary on oocyte morphological quality and blastocyst rates, COCs from Ipsi and Contra follicles were collected (6 biological replicates). COCs groups originating from follicles with different intrafollicular P4 concentrations showed a similar rate of viable and non-viable oocytes (Table 1). Furthermore, when viable oocytes were submitted to *in vitro* embryo production, no difference in blastocyst rate (number of blastocysts on day 7 of development/number of presumptive zygotes placed in *in vitro* culture) was detected (Fig 2). Therefore, in this experimental model CL presence does not impact external quality parameters of oocyte quality, and the blastocyst rates.

**Table 1:** Representation of oocyte recovery rate from iFF and cFF according to morphological parameters

**Group**	**Oocytes of ipsilateral follicles (%)**	**Oocytes of contralateral follicles (%)**	***P*-value**
GI	94 (37.28)	96 (44.29)	0.10
GII	40 (15.44)	28 (12.63)	0.43
GII	60 (22.81)	55 (25.33)	0.66
Viable oocytes	194 (75.54)	179 (82.26)	0.06
Non-viable oocytes	60 (24.45)	36 (17.73)	0.06

G1- Oocytes grade 1. G2- Oocytes grade 2. G3- Oocytes grade 3. Viable oocyte- G1, G2 and G3. These values represent the mean rates calculated based on the total number of recovered oocytes

### The CL presence in the ovary increases ADAMTS-1 and AGO2 in follicular cells

**Fig. 3:**
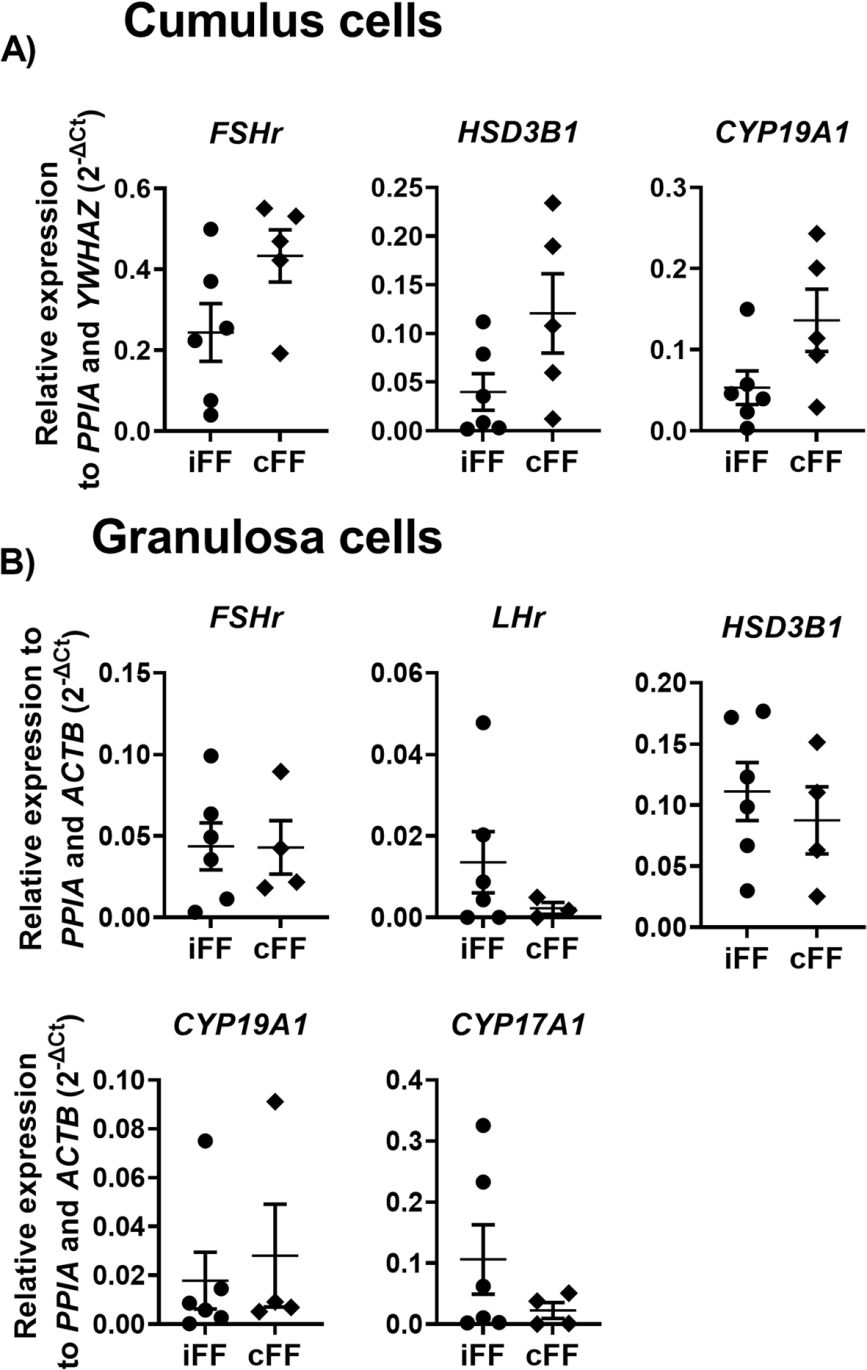
Relative expression levels of steroidogenesis related-genes. (**A**) Cumulus cells and (**B**) granulosa cells expression levels of genes related to ovarian steroidogenesis pathway, collected from follicles ipsilateral and contralateral to the corpus luteum. Points represent the value of each replicate. The black line between the error bars represents the group means and the error bars represent the standard error of the means. Different letters indicate statistical differences (*p*<0.05)

**Fig. 4:**
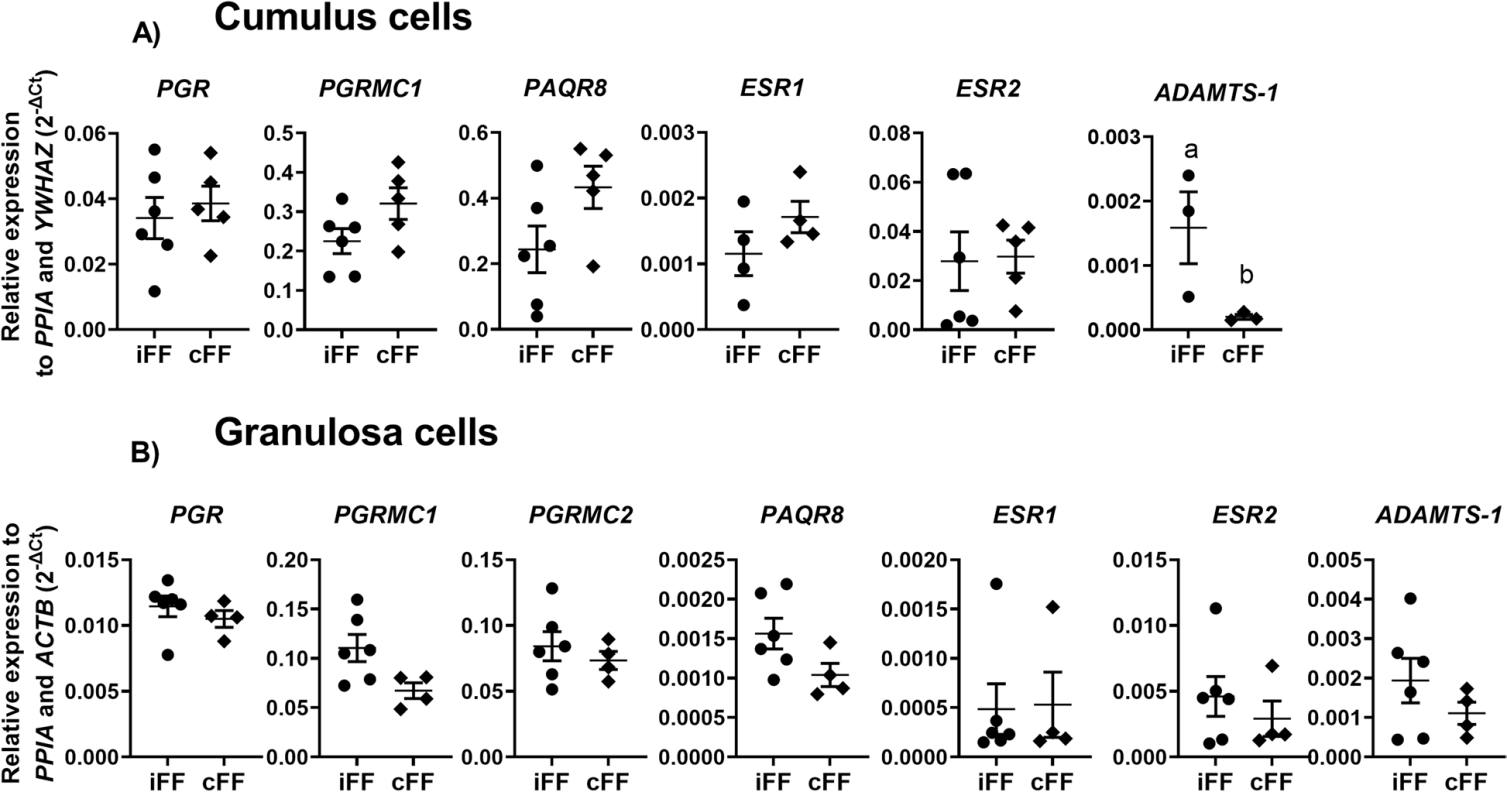
Relative expression levels of oocyte maturation pathway related-genes. (**A**) Cumulus cells and (**B**) granulosa cells expression levels of genes related to oocyte maturation pathway collected from follicles ipsilateral and contralateral to the corpus luteum. Points represent the value of each replicate. The black line between the error bars represents the group means and the error bars represent the standard error of the means. Different letters indicate statistical differences (*p*<0.05)

**Fig. 5:**
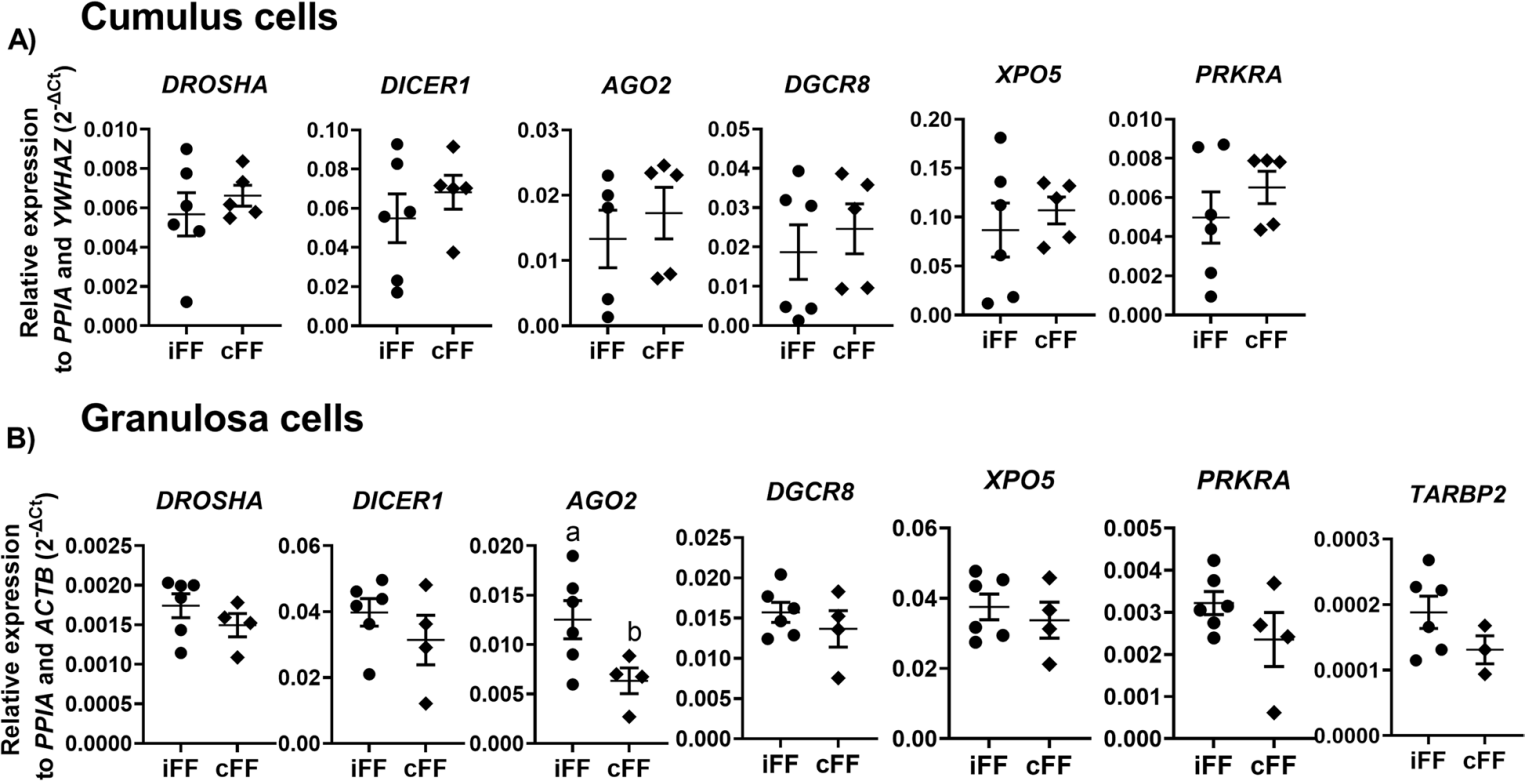
Relative expression levels of miRNA biosynthesis genes. (**A**) Cumulus cells and (**B**) granulosa cells expression levels of genes related to miRNA biosynthesis pathway collected from follicles ipsilateral and contralateral to the corpus luteum. Points represent the value of each replicate. The black line between the error bars represents the group means and the error bars represent the standard error of the means. Different letters indicate statistical differences (*p*<0.05)

To evaluate the effect of different follicle origins regarding the different cell compartments we analyzed genes related to ovarian steroidogenesis, oocyte maturation, and miRNAs biosynthesis pathways in follicular cells (granulosa and cumulus). No differences were observed in the ovarian steroidogenesis pathway, in cumulus and granulosa cells obtained from ipsilateral and contralateral ovaries (Fig 3A and B). Regarding the oocyte maturation pathway in cumulus cells, high *ADAMTS-1* levels were observed in the ipsilateral compared to the contralateral group (*p=* 0.012) (Fig 4A). In granulosa cells, all genes related to oocyte maturation pathway presented similar levels in both groups (Fig 4B). Finally, no differences were observed in miRNA biosynthesis-associated genes in cumulus cells between groups (Fig 5A). However, *AGO2* was upregulated in granulosa cells obtained from ipsilateral (Fig 5B) compared to contralateral follicles (*p*= 0.046). Based on these results, we hypothesized that the presence of a CL within the ovary and consequently the intrafollicular P4 concentrations can modify biological processes related to extracellular matrix remodeling (*ADAMTS-1*) and miRNA biosynthesis (*AGO2)* in follicular cells during follicular development.

### Follicular fluid sEVs are morphologically and numerically similar regardless of CL presence in the ovary

**Fig. 6:**
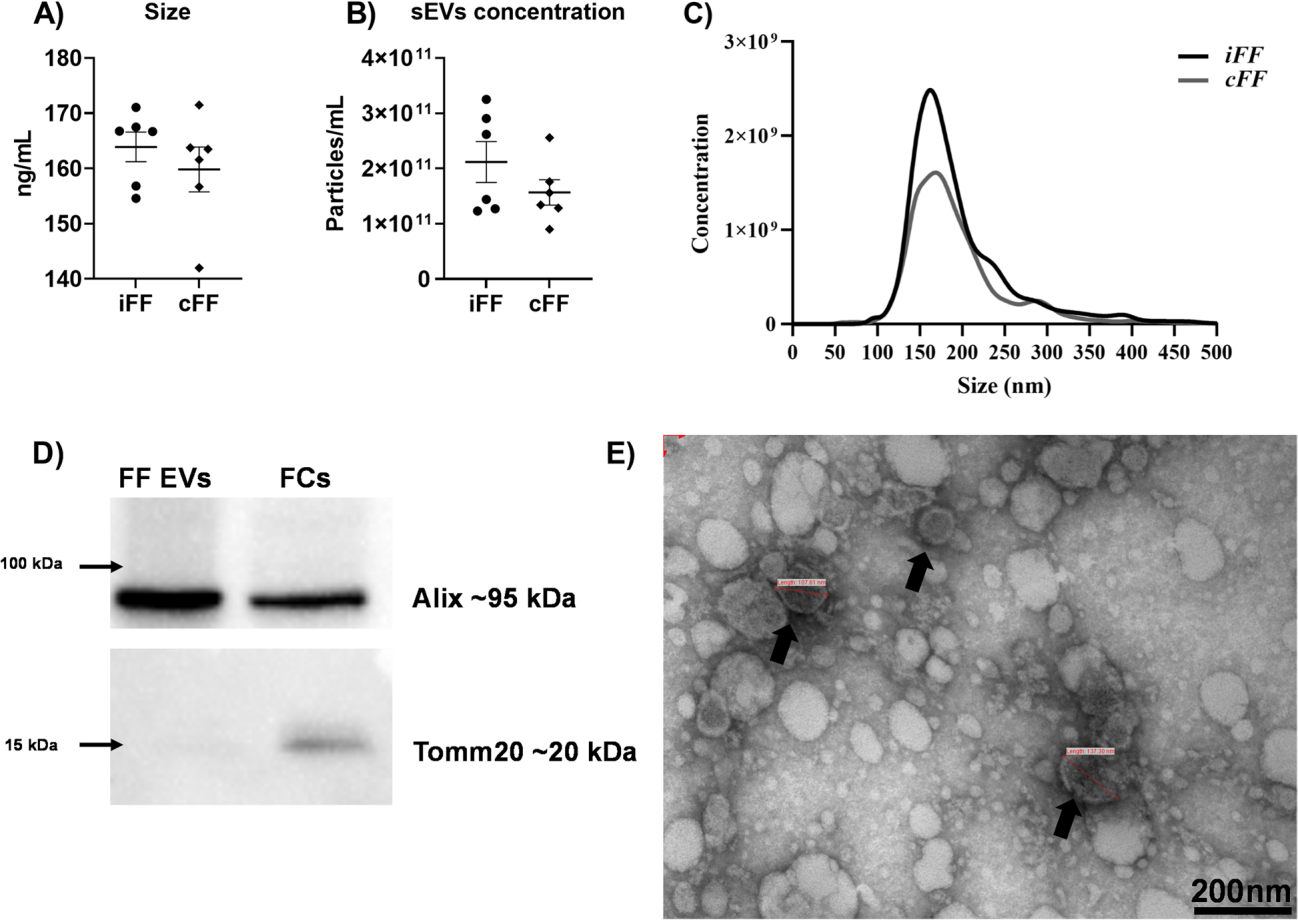
Characterization of small extracellular vesicles (sEVs) from follicular fluid (FF). (**A**). Size and (**B**) concentration of FF sEVs analyzed (n=6) by nanotracking analysis equipment. The black line between the error bars represents the group means and the error bars represent the standard error of the means. (**C**) Distribution of size and particles/Ml. (**D**) Western blotting analysis of Alix and Tomm20 in FF sEVs and follicular cells (FCs). The images demonstrated Alix presence, an accessory protein of the ESCRT complex, in sEVs and FCs. Tomm20, a translocase protein of the outer mitochondrial membrane complex, was not detected in sEVs, only in FCs. Western blot images were cropped and the entire membrane image can be found in the supplementary figure S1. (**E**) Transmission electron microscopy of FF sEVs isolated from follicular fluid, demonstrating the cup-shaped phenotype of the nanoparticles (black arrows)

To investigate the effects of CL presence in FF sEVs characteristics, sEVs obtained from iFF and cFF were isolated and analyzed by NTA. iFF sEVs presented similar size (163.89 ± 1.04 nm; Fig 6A) and particle concentration (2.11 x 10^11^ ± 1.23 x 10^5^ particles/mL; Fig 6B) when compared to cFF sEVs (158.0 ± 1.28 nm; 1.17 x 10^11^ ± 9.70 x 10^4^ particles/mL). The average size and concentration distribution can be observed in Fig. 6C. Next, to investigate the isolation protocol efficiency, additional FF sEVs samples were used for western blot and TEM analysis. The presence of Alix, as well the absence of Tomm20 protein in FF small EVs by western blot confirmed the presence of sEVs in the pellet and the absence of cell contamination, respectively (Fig 6D. Supplementary Figure S2). Images of FF small EVs using transmission electron microscopy demonstrated nanoparticles with cup-shape morphology and <200 nm in diameter (Fig 6E). These finds support the idea that the follicle microenvironment with increased P4 concentration does not alter FF sEVs morphology and secretion patterns. Besides, the isolation protocol can enrich the pellet without coprecipitation of cell debris, due to the absence of Tomm20 in FF sEVs, a protein exclusively present in mitochondria.

### Corpus luteum presence affects FF small EVs miRNA contents

**Fig. 7:**
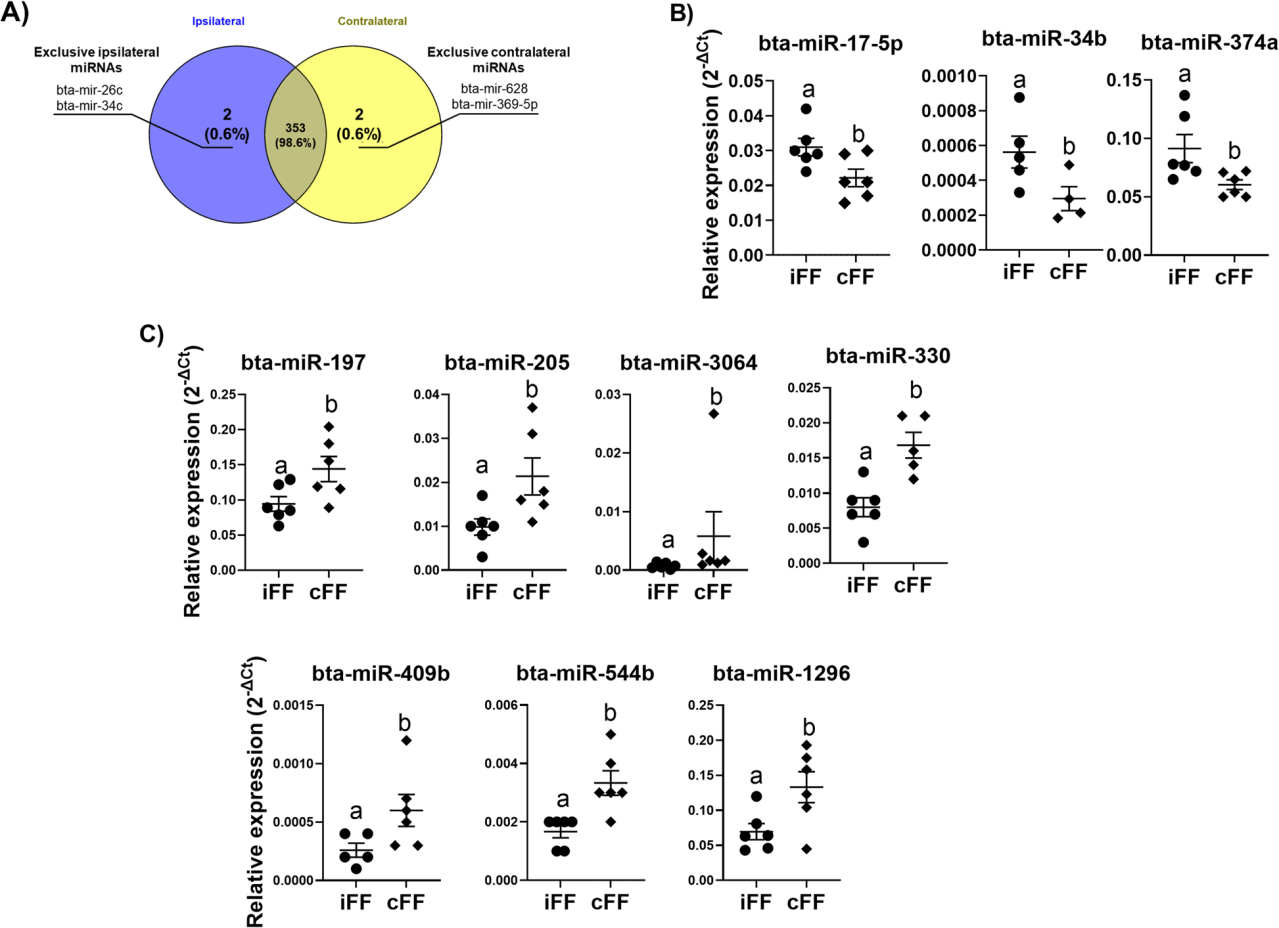
Follicular fluid small extracellular vesicles miRNA profile. (**A**) Venn diagram demonstrating the number of miRNAs detected in each group. (**B**) Three miRNAs were upregulated in iFF small EVs. (**C**) Seven miRNAs were upregulated in cFF small ​EVs. Points represent the value of each replicate. The black line between the error bars represents the group means and error bars represent the standard error of the means and error bars represent the standard error of the means (SEM). Different letters indicate statistical differences

To evaluate the miRNA contents of sEVs from ovarian follicular fluid in the same estrous cycle stage with or without the CL presence, a total of 383 bovine miRNAs were analyzed by RT-qPCR. Three hundred and fifty-three miRNAs were detected in both groups (Fig 7A). MiRNAs in iFF were detected being two (miR-26c and miR-34c) exclusively (Fig 7A) and three upregulated (miR-17-5p, miR-34b, and miR-374a; Fig 7B). MiRNAs in cFF were two (bta-miR-628, and bta-miR-369-5p) exclusive (Fig 7A) and seven upregulated (miR-197, miR-205, miR-3064, miR-330, miR-544b, miR-409b, and miR-1296; Fig 7C). A full list of detected miRNAs and their raw cycle threshold levels are shown in supplementary Table S3. Therefore, these results demonstrate that different intrafollicular P4 concentration caused by CL presence or absence within the ovary induces changes in FF sEVs miRNAs contents.

### Biological pathways predicted to be regulated by miRNAs of FF small EVs differ regarding follicles origin in ovaries with or without the CL presence

**Fig. 8:**
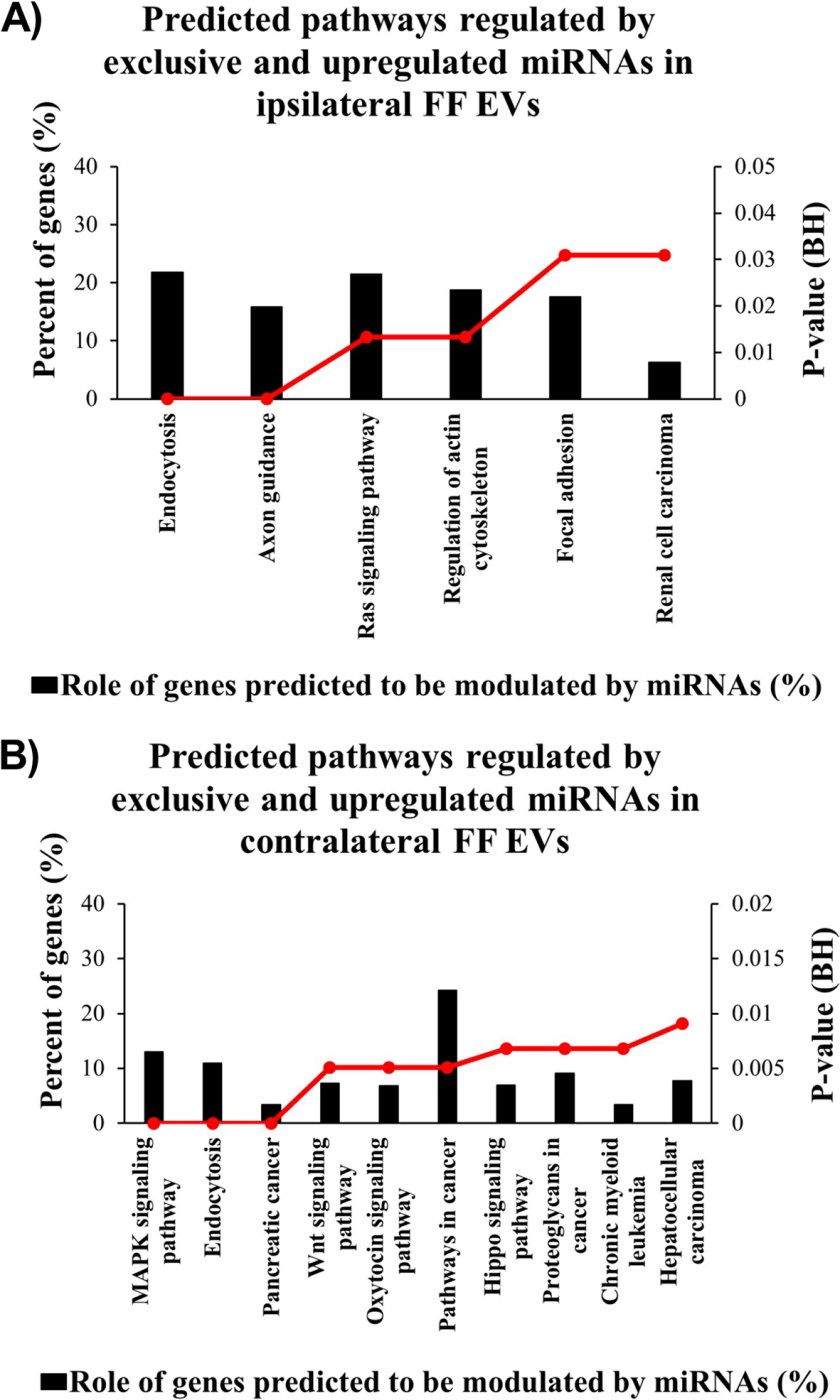
Enrichment analysis of predicted pathways regulated by differently and exclusive expressed miRNAs present in small extracellular vesicles miRNAs from follicular fluid. (**A**) Predicted pathways modulated by two (bta-miR-34c; bta-miR-26c) exclusive sEVs miRNAs and three sEVs miRNAs (bta-miR-17-5p, bta-miR-34b, and bta-miR-374a) upregulated in ipsilateral (high P4) to the corpus luteum follicles. (**B**) Predicted pathways modulated by two (bta-miR-628; bta-miR-369-5p) exclusive sEVs miRNAs and seven sEVs miRNAs (bta-miR-197, bta-miR-205, bta-miR-3064, bta-miR-330, bta-miR-544b, bta-miR-409b, and bta-miR-1296) upregulated in contralateral (low P4) to the corpus luteum follicles. Graphs A (all pathways) and B (first 10 pathways) represent the pathways adjusted by the BH value (*p*<0.05) with a significant level. The left Y-axis values represent the percent of genes modulated by the exclusive and upregulated miRNAs, calculated by the ratio of all genes present in each pathway. The right Y-axis represents the BH value of the pathways predicted with miRWalk 3.0 software

To investigate predicted biological mechanisms regulated by miRNAs differently and exclusively expressed in sEVs from iFF or cFF, we conducted the bioinformatics analysis using MirWalk 3.0 version. For this analysis, were considered the enrichment pathways with statistical significance ranked by BH value (p<0.05). Our results demonstrated that upregulated and exclusively expressed miRNAs from iFF sEVs were predicted to modulate pathways such as endocytosis, axon guidance, Ras signaling, regulation of actin cytoskeleton, focal adhesion, and renal cell carcinoma (Fig 8A). Nevertheless, upregulated and exclusively expressed miRNAs in cFF sEVs were predicted to modulate with BH <0.05 more than 30 pathways. The first ten pathways are related to Endocytosis, Pancreatic cancer, MAPK signaling pathway, Wnt signaling pathway, Oxytocin signaling pathway, Pathways in cancer, Proteoglycans in cancer, Chronic myeloid leukemia, Axon guidance, and Hepatocellular carcinoma (Fig 8B). Thus, these miRNAs present in sEVs from iFF or cFF are predicted to regulate pathways important to follicular development, which could impact oocyte competence acquisition and consequently embryo development capacity.

## Discussion

In this study, we used CL presence or absence in the ovary as an approach to evaluate local P4 influence (intrafollicular) on oocyte quality and competence, gene expression of specific targets in cumulus and granulosa cells, as well as FF sEVs miRNAs profile. The main results demonstrate that CL presence in the ovary increased P4 concentration within the follicular environment. Furthermore, ipsilateral, or contralateral cumulus and granulosa cells differentially expressed genes related to oocyte maturation and miRNA biosynthesis, respectively. Additionally, iFF and cFF sEVs miRNAs were differently expressed, and they are predicted to modulate distinct biological pathways. To our knowledge, these are the first results demonstrating that CL presence in the ovary at the same estrous cycle stage alters the intrafollicular concentration of P4 and impacts follicular cells communication within the follicular environment in bovine.

During estrous cycle stages, P4 oscillations in the intrafollicular environment [26, 27] suggest that its modulation occurs at stage-dependent manner [7]. Our results showed that growing follicles ipsilateral to the CL were under the influence of high P4 concentration. We found around three times higher P4 concentrations in iFF than in cFF. It is well known that the CL presence causes a gradient of P4 concentration along the reproductive tract [28]. Therefore, the CL presence in the ovary contributes to the high P4 intrafollicular concentration [11]. To investigate the influence of CL presence in the intrafollicular environment, pools of follicles ipsi and contralateral to the CL, exposed to different intrafollicular P4 concentrations, were used to obtain the COCs and follicular fluid. In our study oocyte quality grade was not affected by CL presence. Oocyte morphological parameters are a suitable tool to identify signs of quality and predicted embryo potential development [29]. The corpus luteum presence in the ovary has previously been documented to reduce the expansion of cumulus cells and blastocysts rate [10, 11]. In our experimental model, unlike in other studies [9, 11], only 3-6 mm follicles from ovaries in the diestrus stage of the estrous cycle were utilized, which could explain some of the differences. Base on our results of oocyte morphological parameters we can suggest that oocytes were at the same follicular development stage in both experimental groups. Furthermore, intrafollicular P4 may regulate biological mechanisms within other follicular compartments that may not alter external oocyte quality parameters or blastocyst rate but possibly alter embryo quality or even pregnancy rate, although we did not test this in the present manuscript.

To investigate P4 effects in follicular cells, genes related to oocyte maturation and miRNA biosynthesis were analyzed. In the oocyte maturation pathway, no differences were observed in the expression of P4 receptors by follicular cells. During IVM, the P4 supplementation resulted in a dose-dependent alteration in the expression of both nuclear and membrane receptors in cumulus cells [30]. We hypothesize that exposure to P4 in contralateral follicles, albeit at lower levels, may still be substantial and, given its chronic exposition nature, could have also induced the expression of P4 receptors in this group. To investigate this hypothesis, assessing individual effects on cumulus cells after P4 supplementation during IVM may provide a means to clarify these findings.

However, this variation in P4 concentration was capable of enhancing the expression of ADAMTS-1 transcripts in cumulus cells from IFF groups, a gene associated with oocyte maturation. *ADAMTS-1* is a metalloproteinase involved in extracellular matrix remodeling through protease activity [31]. *ADAMTS-1* knockdown in cumulus cells repressed COC maturation by reduction of cumulus cell expansion, and consequently oocyte progression from GV to MII stage [32]. Then, we postulate that high levels of *ADAMTS-1* in cumulus cells from 3-6mm iFF may trigger cumulus extracellular matrix remodeling without additional maturation induction stimuli. Additionally, we detected higher *AGO2* expression in granulosa cells from iFF compared to cFF. *AGO2* expression was described to be increased in the ampulla region compared to the isthmus region of the oviduct, demonstrating that CL proximity and high P4 can modulate this gene [33]. AGO2 is a pivotal protein in the silencing complex formation that regulates the final step of miRNA induced gene silencing [34]. This suggests that P4 can regulate part of the miRNA biogenesis pathway and could possibly be caused by a dysregulation of their biogenesis in granulosa cells.

To evaluate the effects of P4 on FF sEVs content we evaluated their miRNA profile. Our results demonstrate that iFF sEVs presented five, while cFF sEVs nine differently and exclusively expressed miRNAs. MiR-34c an exclusively detected miRNA in iFF, is associated with lower ovarian cancer cell proliferation [35]. Besides, miR-17-5p upregulated in iFF is related to ovarian cancer cell that undergo epithelial-mesenchymal transition (EMT) [36]. During follicular cell development, both events: decreased proliferation and EMT, are required for follicular cell development culminating with follicle ovulation [37]. Besides, functional enrichment analysis demonstrated that these miRNAs in iFF sEVs were predicted to modulate pathways related to cell communication process as endocytosis, axon guidance, regulation of actin cytoskeleton, and focal adhesion.

On the other hand, the exclusives miRNA in cFF, bta-miR-628 is related to prostatic cancer proliferation [38], and bta-miR-369-5p is related to decreased adipocyte differentiation in EMT [39]. In addition, the upregulation of miR-205, in cFF could impact granulosa cells, which was previously identified to express PTX3 as a putative target gene [40]. PTX3 protein regulates cumulus cell expansion through matrix stability during oocyte maturation [41]. Finally, miR-205 was downregulated in mature oocytes compared to the immature oocytes [42]. Additionally, differently expressed and exclusively detected miRNAs in the cFF sEVs modulate pathways related to signal transduction such as MAPK signaling pathway, and hippo signaling pathway. These results suggest that iFF sEVs transfer signals capable of inducing follicular cell maturation progression (cell differentiation), while cFF sEVs carry messages related to cell proliferation.

In conclusion, the present study showed that CL presence in the ovary is associated with high intrafollicular P4 concentrations during the same estrous cycle stage. Although we have not detected direct effects on COC quality grade and blastocyst rate, *ADAMTS-1* transcript related to extracellular matrix remodeling and *AGO2* transcript related to gene silencing were upregulated in cumulus and granulosa cells of iFF group, which could be inducing processes related to the progression of oocyte maturation in FF sEVs miRNAs. Overall, this study showed that sEVs contents from different P4 environments may regulate follicular environment and consequently alters oocyte developmental competence through precocious induction of oocyte maturation cascade. These results suggest that modulation of P4 intrafollicular levels could act as a possible functional approach to improve oocyte quality and embryo development. Nonetheless, it is essential additional studies to demonstrate the functional effects of P4 or sEVs of iFF and cFF on the oocyte during maturation both in vitro and in vivo.

### Supplementary Information

**Supplementary Material 1.** 

**Supplementary Material 2.** 

**Supplementary Material 3.** 

**Supplementary Material 4.** 

## Data Availability

The datasets used and/or analyzed during the current study are available from the corresponding author on reasonable request.
