# Supplementary figures and images for: Corpus luteum presence in the bovine ovary increase intrafollicular progesterone concentration: consequences in follicular cells gene expression and follicular fluid small extracellular vesicles miRNA contents
Source: J Ovarian Res. 2024 Mar 18;17:65. doi: 10.1186/s13048-024-01387-3 (PMC10946200; doi:10.1186/s13048-024-01387-3)

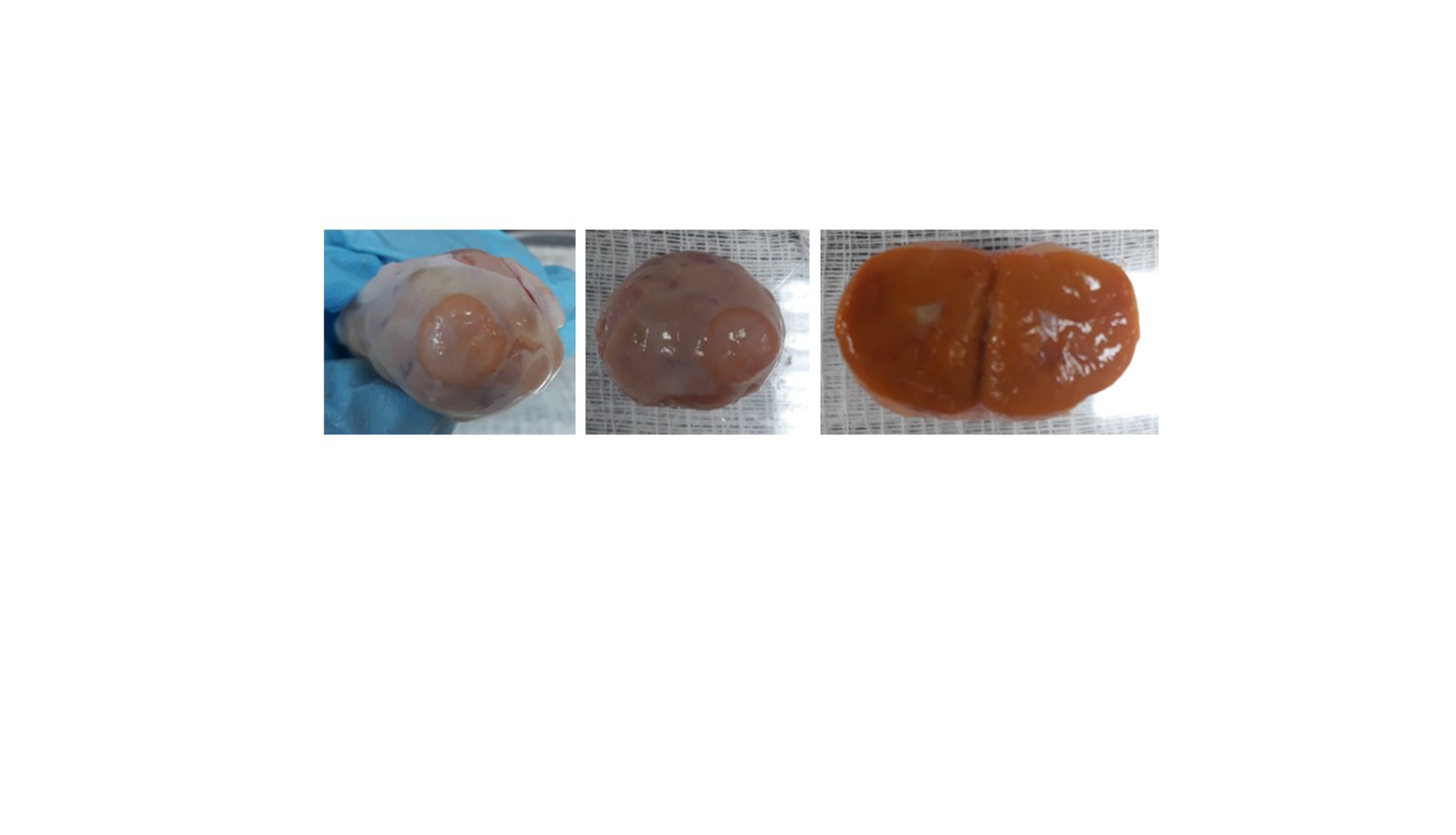

Supplement: Supplementary file 1 — Supplementary Material 1. [file 13048_2024_1387_MOESM1_ESM.tif]

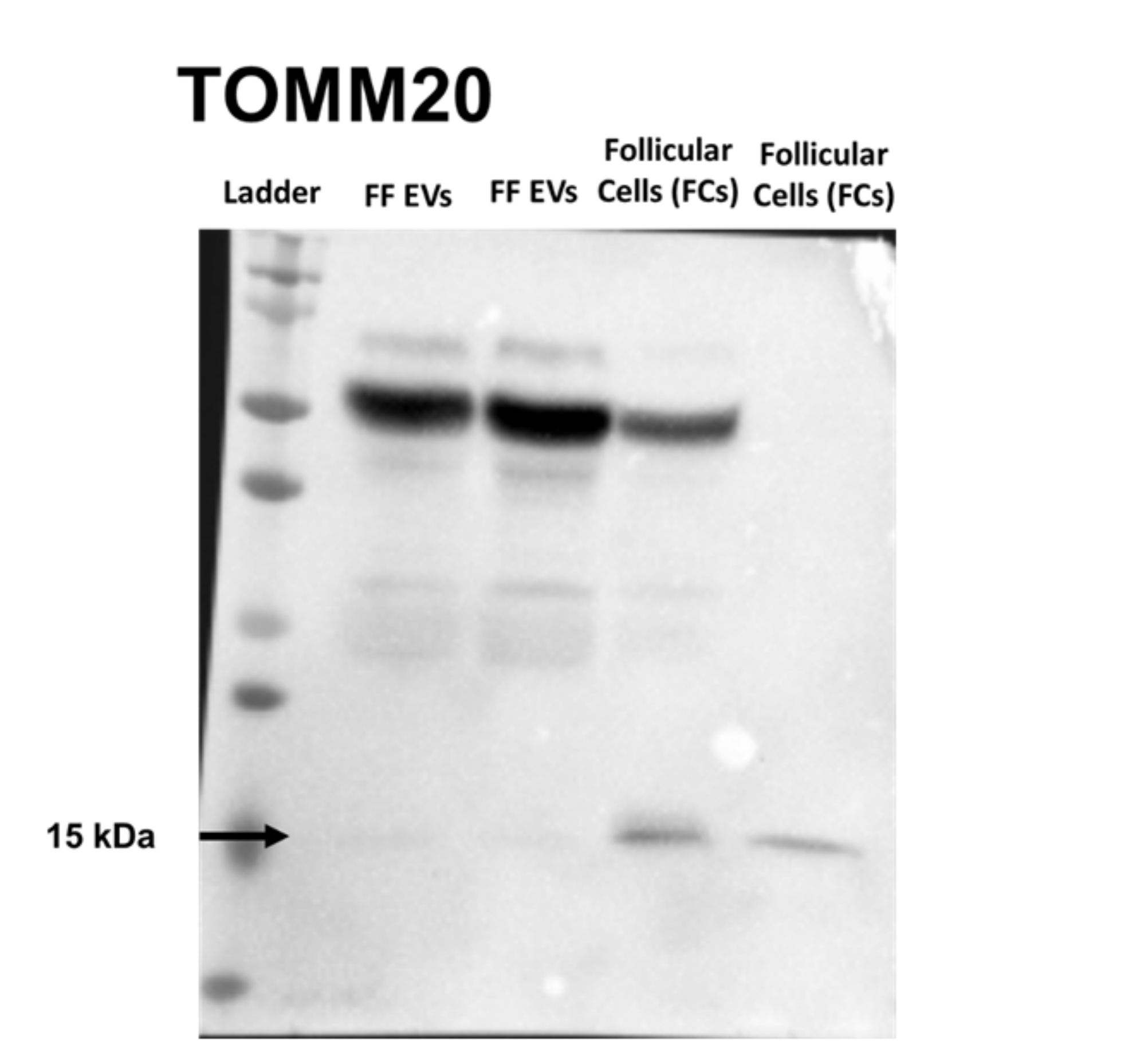

Supplement: Supplementary file 2 — Supplementary Material 2. [file 13048_2024_1387_MOESM2_ESM.zip › Supplemental Figura S2_TOMM20.tif]

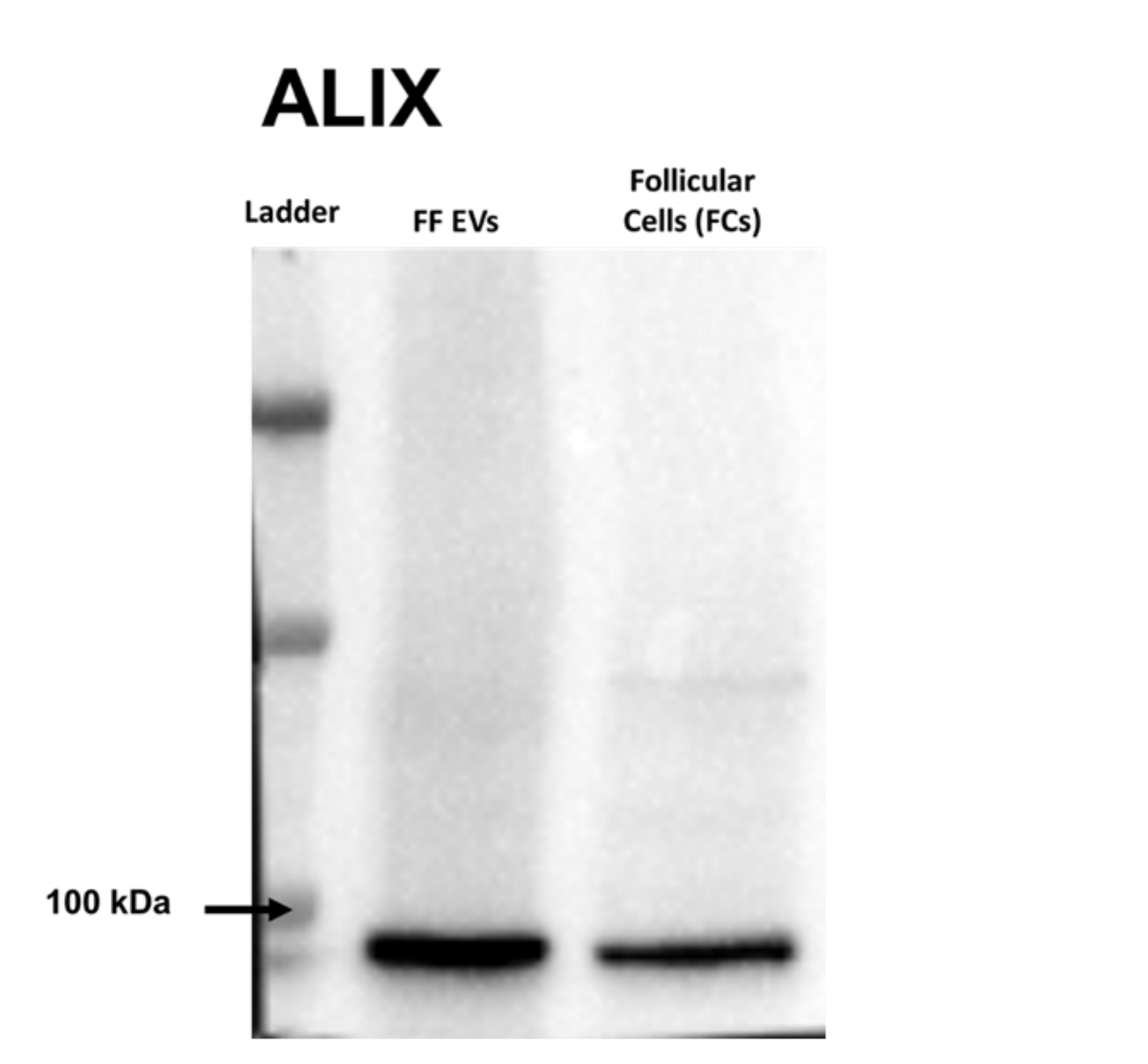

Supplement: Supplementary file 2 — Supplementary Material 2. [file 13048_2024_1387_MOESM2_ESM.zip › Supplemental Figure S2_Alix.tif]
