# Supplementary material for: Corpus luteum presence in the bovine ovary increase intrafollicular progesterone concentration: consequences in follicular cells gene expression and follicular fluid small extracellular vesicles miRNA contents
Source: J Ovarian Res. 2024 Mar 18;17:65. doi: 10.1186/s13048-024-01387-3 (PMC10946200; doi:10.1186/s13048-024-01387-3)
Supplement: Supplementary file 3 — Supplementary Material 3. [file 13048_2024_1387_MOESM3_ESM.docx]

**Supplementary material**

**Supplementary Table S1.** Primers sequences of genes analyzed by RT-PCRq

| **Gene** | **Gene name** | **Primer** | **Sequence 5' - 3'** | **Reference** |
| --- | --- | --- | --- | --- |
| *FSHr* | Follicle Stimulating Hormone Receptor | *F* | GGAGGCGGCAAACCTCTGA | Designed by authors |
|  |  | *R* | TCTCTGACCCCTAGCCTGAGT |  |
| *LHr* | Luteinizing Hormone Receptor | *F* | TCTCAGAGTGATTCCCTGGAAAAGA | Designed by authors |
|  |  | *R* | CAGCCTCAATGTGCACCAGG |  |
| *3b-HSD* | 3b-hydroxysteroid dehydrogenase | *F* | GCCACAATCTGACCGCATCG | Designed by authors |
|  |  | *R* | CACCCTGCCATGGCCAAATC |  |
| *ADAMTS-1* | Disintegrin and metalloproteinase with thrombospondin motifs 1 | *F* | CGGGACACCGAACAGGAACT | Designed by authors |
|  |  | *R* | AGGTCACTGCCGTGGAACTC |  |
| *CYP19A1* | Cytochrome P450 Family 19 Subfamily A Member 1 | *F* | TGTGGACGTGTTGACCCTCA | Designed by authors |
|  |  | *R* | ACCACGATGGCACTTTCATCC |  |
| *CYP17A1* | prostaglandin-endoperoxide synthase 2 | *F* | AACCATCAGTGACCGGAACC | Designed by authors |
|  |  | *R* | TAAGGTCGCCAATGCTGGAG |  |
| *PGR* | Genomic P4 receptor | *F* | AGGGCAATGGAAGGACAGCA | Designed by authors |
|  |  | *R* | GCGACATGCTGGGCAGTTTT |  |
| *PAQR8* | Nongenomic membrane progestin receptors | *F* | TGTAGCCTTGCGAGACACAG | Designed by authors |
|  |  | *R* | CGGAGGCCACAGTCAGATTT |  |
| *PGRMC1* | Nongenomic P4 receptor membrane components 1 | *F* | CTGGGACTCTCAGTTCACTTTCAA | Designed by authors |
|  |  | *R* | TGGCTCCTCCTTGTCTGAGT |  |
| *PGRMC2* | Nongenomic P4 receptor membrane components 2 | *F* | CCAGGGGAAGAACCGTCAGAA | Designed by authors |
|  |  | *R* | TGAAGGCCCCTGACTTTGGTT |  |
| *ESR1* | Estrogen receptor 1 | *F* | TCCCTTCCTTCTAACTGTCTCAGC | Designed by authors |
|  |  | *R* | GCCGGCCACCCTGCT |  |
| *ESR2* | Estrogen receptor 2 | *F* | CGTCAGGCACGCCAGTAACA | Designed by authors |
|  |  | *R* | TTGTTGCCGCGAAGTGTGTG |  |
|  |  | *R* | CCAGATAGGCACCCAGGG |  |
| *DROSHA* | Drosha Ribonuclease III | *F* | AAGGCAGTGCATGTCACAGAA | doi.org/10.3390/ijms22020953 |
|  |  | *R* | GCTGGGAGGTTCGTATTGGT |  |
| *DGCR8* | DGCR8 Microprocessor Complex Subunit | *F* | TCATCAACCCCAACGGGAAG | Designed by authors |
|  |  | *R* | TCACTTGGGTTCTCGCACTC |  |
| *DICER1* | Dicer1 ribonuclease typo III | *F* | TCACGATCAACACGGCCATT | Designed by authors |
|  |  | *R* | TTGGGGGACCAACAATGGAG |  |
| *AGO2* | Argonaute RISC catalytic component 2 | *F* | GTTTATGCCGAGGTCAAGCG | Designed by authors |
|  |  | *R* | CAGCAGGATGTTGTTCACGC |  |
| *XPO5* | Exportin 5 | *F* | TCATCAACCCCAACGGGAAG | Designed by authors |
|  |  | *R* | TCACTTGGGTTCTCGCACTC |  |
| *PRKRA* | Protein Activator Of Interferon Induced Protein Kinase | *F* | GCAAGTATTTGCTTTGCAGTTCC | Designed by authors |
|  |  | *R* | CTCCCTCCTGGGAAAGGGTA |  |
| *TARBP2* | TARBP2 Subunit Of RISC Loading Complex | *F* | CTAGCTCGTCGGCGGTGTAT | Designed by authors |
|  |  | *R* | CATTTGCTCTATACTGGGCAGC |  |
| *PPIA* | peptidylprolyl isomerase A | *F* | GCCATGGAGCGCTTTGG | doi:10.1371/journal.pone.0145321 |
|  |  | *R* | CCACAGTCAGCAATGGTGATCT |  |
| *YWHAZ* | Tyrosine 3-monooxygenase/tryptophan 5-monooxygenase activation protein zeta | *F* | GCATCCCACAGACTATTTCC | doi.org/10.1371/journal.pone.018504 |
|  |  | *R* | GCAAAGACAATGACAGACCA |  |
| *ACTB* | Actin Beta | *F* | CAGCAGATGTGGATCAGCAAGC | doi.org/10.1371/journal.pone.018504 |
|  |  | *R* | AACGCAGCTAACAGTCCGCC |  |

**Supplementary Table S2**. MicroRNA forward primerZ sequences used in quantitative RT-PCRq

| **MiRNA** | **Sequence** | **MiRNA** | | **Sequence** | |
| --- | --- | --- | --- | --- | --- |
| bta-let-7a-3p | CTATACAATCTACTGTCTTTC | bta-miR-340 | | TCCGTCTCAGTTACTTTATAGCC | |
| bta-miR-103 | AGCAGCATTGTACAGGGCTATGA | bta-miR-365-3p | | TAATGCCCCTAAAAATCCTTAT | |
| bta-let-7a-5p | TGAGGTAGTAGGTTGTATAGTT | bta-miR-342 | | TCTCACACAGAAATCGCACCCATCT | |
| bta-miR-105a | TCAAATGCTCAGACTCCTGTGGT | bta-miR-365-5p | | AGGGACTTTTGGGGGCAGATGTG | |
| bta-let-7b | TGAGGTAGTAGGTTGTGTGGTT | bta-miR-345-3p | | CCTGAACTAGGGGTCTGGAG | |
| bta-miR-105b | TCAAATGCTCAGACTCCTTGGT | bta-miR-367 | | GAATTGCACTTTAGCAATGGTGA | |
| bta-let-7c | TGAGGTAGTAGGTTGTATGGTT | bta-miR-345-5p | | GCTGACTCCTAGTCCAGTGCT | |
| bta-miR-106a | AAAAGTGCTTACAGTGCAGGTA | bta-miR-369-3p | | AATAATACATGGTTGATCTTT | |
| bta-let-7d | AGAGGTAGTAGGTTGCATAGTT | bta-miR-346 | | TGTCTGCCCGCATGCCTGCCTCT | |
| bta-miR-106b | TAAAGTGCTGACAGTGCAGAT | bta-miR-369-5p | | ATCGACCGTGTTATATTCGC | |
| bta-let-7e | TGAGGTAGGAGGTTGTATAGT | bta-miR-34a | | TGGCAGTGTCTTAGCTGGTTGT | |
| bta-miR-107 | AGCAGCATTGTACAGGGCTATC | bta-miR-370 | | GCCTGCTGGGGTGGAACCTGGT | |
| bta-let-7f | TGAGGTAGTAGATTGTATAGTT | bta-miR-34b | | AGGCAGTGTAATTAGCTGATTG | |
| bta-miR-10a | TACCCTGTAGATCCGAATTTGTG | bta-miR-371 | | AAGTGCCGCCATGTTTTGAGTGT | |
| bta-let-7g | TGAGGTAGTAGTTTGTACAGTT | bta-miR-34c | | AGGCAGTGTAGTTAGCTGATTG | |
| bta-miR-10b | TACCCTGTAGAACCGAATTTGTG | bta-miR-374a | | TTATAATACAACCTGATAAGTG | |
| bta-let-7i | TGAGGTAGTAGTTTGTGCTGTT | bta-miR-361 | | TTATCAGAATCTCCAGGGGTAC | |
| bta-miR-122 | TGGAGTGTGACAATGGTGTTTG | bta-miR-374b | | ATATAATACAACCTGCTAAGTG | |
| bta-miR-1 | TGGAATGTAAAGAAGTATGTAT | bta-miR-362-3p | | AACACACCTATTCAAGGATTC | |
| bta-miR-124a | TAAGGCACGCGGTGAATGCCAAG | bta-miR-375 | | TTTTGTTCGTTCGGCTCGCGTGA | |
| bta-miR-100 | AACCCGTAGATCCGAACTTGTG | bta-miR-362-5p | | AATCCTTGGAACCTAGGTGTGAGT | |
| bta-miR-124b | TAAGGCACGCGGTGAATGCCAAG | bta-miR-376a | | ATCATAGAGGAAAATCCACGT | |
| bta-miR-101 | TACAGTACTGTGATAACTGAA | bta-miR-363 | | ATTGCACGGTATCCATCTGCG | |
| bta-miR-125a | TCCCTGAGACCCTTTAACCTGTG | bta-miR-376b | | ATCATAGAGGAAAATCCATGTT | |
| bta-miR-125b | TCCCTGAGACCCTAACTTGTGA | bta-miR-376c | | GTGGATATTCCTTCTATGTTTA | |
| bta-miR-133b | TTTGGTCCCCTTCAACCAGCTA | bta-miR-382 | | GAAGTTGTTCGTGGTGGATTCG | |
| bta-miR-126-3p | CGTACCGTGAGTAATAATGCG | bta-miR-376d | | ATCATAGAGGAAAATCCACAT | |
| bta-miR-133c | ATTTGGTTCCATTTTACCAGC | bta-miR-383 | | AGATCAGAAGGTGATTGTGGCT | |
| bta-miR-126-5p | CATTATTACTTTTGGTACGCG | bta-miR-376e | | AACATAGAGGAAAATCCACATT | |
| bta-miR-134 | TGTGACTGGTTGACCAGAGTGG | bta-miR-409a | | AGGTTACCCGAGCAACTTTGCAT | |
| bta-miR-127 | TCGGATCCGTCTGAGCTTGGCT | bta-miR-377 | | ATCACACAAAGGCAACTTTTGT | |
| bta-miR-135a | TATGGCTTTTTATTCCTATGTGA | bta-miR-409b | | GGGGTTCACCGAGCAACATTC | |
| bta-miR-128 | TCACAGTGAACCGGTCTCTTT | bta-miR-378 | | ACTGGACTTGGAGTCAGAAGGC | |
| bta-miR-135b | TATGGCTTTTCATTCCTATGTGA | bta-miR-410 | | AATATAACACAGATGGCCTGT | |
| bta-miR-129 | CTTTTTGCGGTCTGGGCTTGCT | bta-miR-378b | | ACTTGACTTGGAGTCAGAAGGC | |
| bta-miR-136 | ACTCCATTTGTTTTGATGATGGA | bta-miR-411a | | ATAGTAGACCGTATAGCGTACG | |
| bta-miR-129-3p | AAGCCCTTACCCCAAAAAGCAT | bta-miR-378c | | ACTGGACTTGGAGTCAGAAGT | |
| bta-miR-137 | TTATTGCTTAAGAATACGCGTAG | bta-miR-411b | | TGGTCGACCATAAAACGTACGT | |
| bta-miR-129-5p | CTTTTTGCGGTCTGGGCTTGCT | bta-miR-378d | | CTGGACTTGGAGTCAGAAGACC | |
| bta-miR-138 | AGCTGGTGTTGTGAATCAGGCCG | bta-miR-411c-3p | | TGTATGTCAACTGATCCACAGT | |
| bta-miR-130a | CAGTGCAATGTTAAAAGGGCAT | bta-miR-379 | | TGGTAGACTATGGAACGTAGG | |
| bta-miR-139 | TCTACAGTGCACGTGTCTCCAGT | bta-miR-411c-5p | | GGTTGATCAGAGAACATACATT | |
| bta-miR-130b | CAGTGCAATGATGAAAGGGCAT | bta-miR-380-3p | | TATGTAATGTGGTCCACGTCT | |
| bta-miR-140 | TACCACAGGGTAGAACCACGGA | bta-miR-412 | | ACTTCACCTGGTCCACTAGCTGT | |
| bta-miR-132 | TAACAGTCTACAGCCATGGTCG | bta-miR-380-5p | | TGGTTGACCATAGAACATGCGC | |
| bta-miR-141 | TAACACTGTCTGGTAAAGATGG | bta-miR-421 | | ATCAACAGACATTAATTGGGCGC | |
| bta-miR-133a | TTTGGTCCCCTTCAACCAGCTG | bta-miR-381 | | TATACAAGGGCAAGCTCTCTGT | |
| bta-miR-142-3p | AGTGTTTCCTACTTTATGGATG | bta-miR-423-3p | | AAGCTCGGTCTGAGGCCCCTCAGT | |
| bta-miR-142-5p | CATAAAGTAGAAAGCACTAC | bta-miR-423-5p | | TGAGGGGCAGAGAGCGAGACTTT | |
| bta-miR-151-3p | CTAGACTGAAGCTCCTTGAGG | bta-miR-449c | | AGGCAGTGCATCTCTAGCTGG | |
| bta-miR-143 | TGAGATGAAGCACTGTAGCTCG | bta-miR-424-3p | | CAAAACGTGAGGCGCTGCTAT | |
| bta-miR-151-5p | TCGAGGAGCTCACAGTCTAGT | bta-miR-449d | | GAAGGCTGTGTGCTGTGGAG | |
| bta-miR-144 | TACAGTATAGATGATGTACTAG | bta-miR-424-5p | | CAGCAGCAATTCATGTTTTGA | |
| bta-miR-152 | TCAGTGCATGACAGAACTTGGG | bta-miR-450a | | TTTTGCGATGTGTTCCTAATAT | |
| bta-miR-145 | GTCCAGTTTTCCCAGGAATCCCT | bta-miR-425-3p | | ATCGGGAATGTCGTGTCCGCCC | |
| bta-miR-153 | TTGCATAGTCACAAAAGTGATC | bta-miR-450b | | TTTTGCAATATGTTCCTGAATA | |
| bta-miR-146a | TGAGAACTGAATTCCATAGGTTGT | bta-miR-425-5p | | ATGACACGATCACTCCCGTTGA | |
| bta-miR-154a | TAGGTTATCCGTGTAGCCTTCG | bta-miR-451 | | AAACCGTTACCATTACTGAGTTT | |
| bta-miR-146b | TGAGAACTGAATTCCATAGGCTGT | bta-miR-429 | | TAATACTGTCTGGTAATGCCGT | |
| bta-miR-154b | AGAGGTCTTCCATGGTGCATTCG | bta-miR-452 | | TGTTTGCAGAGGAAACTGAGAC | |
| bta-miR-147 | GTGTGCGGAAATGCTTCTGCTA | bta-miR-431 | | TGTCTTGCAGGCCGTCATGCAGG | |
| bta-miR-154c | AGATATTGCACGGTTGATCTCT | bta-miR-4523 | | GACCGAGAGGGCCTCGGCTGT | |
| bta-miR-148a | TCAGTGCACTACAGAACTTTGT | bta-miR-432 | | TCTTGGAGTAGGTCATTGGGTGG | |
| bta-miR-155 | TTAATGCTAATCGTGATAGGGGT | bta-miR-453 | | AGGTTGTCCGTGGTGAGTTCGCA | |
| bta-miR-148b | TCAGTGCATCACAGAACTTTGT | bta-miR-433 | | ATCATGATGGGCTCCTCGGTGT | |
| bta-miR-15a | TAGCAGCACATAATGGTTTGT | bta-miR-454 | | TAGTGCAATATTGCTTATAGGGT | |
| bta-miR-149-3p | GAGGGAGGGACGGGGGCTGTGC | bta-miR-448 | | TTGCATATGTAGGATGTCCCAT | |
| bta-miR-15b | TAGCAGCACATCATGGTTTACA | bta-miR-455-3p | | GCAGTCCATGGGCATATACACT | |
| bta-miR-149-5p | TCTGGCTCCGTGTCTTCACTCCC | bta-miR-449a | | TGGCAGTGTATTGTTAGCTGGT | |
| bta-miR-16a | TAGCAGCACGTAAATATTGGTG | bta-miR-455-5p | | TATGTGCCTTTGGACTACATC | |
| bta-miR-150 | TCTCCCAACCCTTGTACCAGTGT | bta-miR-449b | | AGGCAGTGTATTGTTAGCTGGC | |
| bta-miR-16b | TAGCAGCACGTAAATATTGGC | bta-miR-483 | | TCACTCCTCTCCTCCCGTCTT | |
| bta-miR-17-3p | ACTGCAGTGAAGGCACTTGT | bta-miR-484 | | TCAGGCTCAGTCCCCTCCCGAT | |
| bta-miR-188 | CATCCCTTGCATGGTGGAGGGT | bta-miR-496 | | TGAGTATTACATGGCCAATCTC | |
| bta-miR-17-5p | CAAAGTGCTTACAGTGCAGGTAGT | bta-miR-485 | | AGAGGCTGGCCGTGATGAATTCG | |
| bta-miR-18a | TAAGGTGCATCTAGTGCAGATA | bta-miR-497 | | CAGCAGCACACTGTGGTTTGTA | |
| bta-miR-181a | AACATTCAACGCTGTCGGTGAGTT | bta-miR-486 | | TCCTGTACTGAGCTGCCCCGAG | |
| bta-miR-18b | TAAGGTGCATCTAGTGCAGTTA | bta-miR-499 | | TTAAGACTTGCAGTGATGTTT | |
| bta-miR-181b | AACATTCATTGCTGTCGGTGGGTT | bta-miR-487a | | AATCATACAGGGACATCCAGT | |
| bta-miR-190a | TGATATGTTTGATATATTAGGT | bta-miR-500 | | TAATCCTTGCTACCTGGGTGAGA | |
| bta-miR-181c | AACATTCAACCTGTCGGTGAGTTT | bta-miR-487b | | AATCGTACAGGGTCATCCACTT | |
| bta-miR-190b | TGATATGTTTGATATTGGGTT | bta-miR-502a | | AATGCACCTGGGCAAGGATTCA | |
| bta-miR-181d | AACATTCATTGTTGTCGGTGGGT | bta-miR-488 | | TTGAAAGGCTGTTTCTTGGTC | |
| bta-miR-191 | CAACGGAATCCCAAAAGCAGCTG | bta-miR-502b | | AATCCACCTGGGCAAGGATTC | |
| bta-miR-182 | TTTGGCAATGGTAGAACTCACACT | bta-miR-489 | | GTGACATCACATATATGGCGAC | |
| bta-miR-192 | CTGACCTATGAATTGACAGCCAG | bta-miR-503-3p | | GGAGTATTGTTTCTGCTGCCCGG | |
| bta-miR-183 | TATGGCACTGGTAGAATTCACTG | bta-miR-490 | | CAACCTGGAGGACTCCATGCTG | |
| bta-miR-193a | GGGACTTTGTAGGCCAGTT | bta-miR-503-5p | | TAGCAGCGGGAACAGTACTG | |
| bta-miR-184 | TGGACGGAGAACTGATAAGGGT | bta-miR-491 | | AGTGGGGAACCCTTCCATGAGG | |
| bta-miR-193a-3p | AACTGGCCTACAAAGTCCCAGT | bta-miR-504 | | AGACCCTGGTCTGCACTCTGTC | |
| bta-miR-185 | TGGAGAGAAAGGCAGTTCCTGA | bta-miR-493 | | TGAAGGTCTACTGTGTGCCAGG | |
| bta-miR-193a-5p | TGGGTCTTTGCGGGCGAGATGA | bta-miR-505 | | CGTCAACACTTGCTGGTTTCCT | |
| bta-miR-186 | CAAAGAATTCTCCTTTTGGGCT | bta-miR-494 | | TGAAACATACACGGGAAACCTC | |
| bta-miR-193b | AACTGGCCCACAAAGTCCCGCTTT | bta-miR-532 | | CATGCCTTGAGTGTAGGACCGT | |
| bta-miR-187 | TCGTGTCTTGTGTTGCAGCCGG | bta-miR-495 | | AAACAAACATGGTGCACTTCTT | |
| bta-miR-194 | TGTAACAGCAACTCCATGTGGA | bta-miR-539 | | GGAGAAATTATCCTTGGTGTGT | |
| bta-miR-195 | TAGCAGCACAGAAATATTGGCA | bta-miR-541 | | TGGTGGGCACAGAATCCGGCCT | |
| bta-miR-200c | TAATACTGCCGGGTAATGATGGA | bta-miR-582 | | TTACAGTTGTTCAACCAGTTACT | |
| bta-miR-196a | TAGGTAGTTTCATGTTGTTGGG | bta-miR-542-5p | | TCGGGGATCATCATGTCACGAG | |
| bta-miR-202 | TTCCTATGCATATACTTCTTT | bta-miR-584 | | TGGTTTGCCTGGGACTGAG | |
| bta-miR-196b | TAGGTAGTTTCCTGTTGTTGGGA | bta-miR-543 | | AAACATTCGCGGTGCACTTCTT | |
| bta-miR-204 | TTCCCTTTGTCATCCTATGCCT | bta-miR-592 | | ATTGTGTCAATATGCGATGATGT | |
| bta-miR-197 | TTCACCACCTTCTCCACCCAGC | bta-miR-544a | | ATTCTGCATTTTTAGCAAGTTC | |
| bta-miR-205 | TCCTTCATTCCACCGGAGTCTG | bta-miR-599 | | GTTGTGTCAGTTTATCAAAC | |
| bta-miR-199a-3p | ACAGTAGTCTGCACATTGGTTA | bta-miR-544b | | ATTCTGCATTTCTAACAAGTTC | |
| bta-miR-206 | TGGAATGTAAGGAAGTGTGTGG | bta-miR-615 | | GGGGGTCCCCGGTGCTCGGATC | |
| bta-miR-199a-5p | CCCAGTGTTCAGACTACCTGTT | bta-miR-545-3p | | ATCAACAAACATTTATTGTGTG | |
| bta-miR-208a | ATAAGACGAGCAAAAAGCTTGT | bta-miR-628 | | ATGCTGACATATTTACTAGAGG | |
| bta-miR-199b | CCCAGTGTTTAGACTATCTGTTC | bta-miR-545-5p | | TCAGTAAATGTTTATTGGATG | |
| bta-miR-208b | ATAAGACGAACAAAAGGTTTGT | bta-miR-631 | | AGACCTGGCTTAGACCTCAGC | |
| bta-miR-199c | TACAGTAGTCTGCACATTGG | bta-miR-551a | | GCGACCCAATCTTGGTTTCCA | |
| bta-miR-20a | TAAAGTGCTTATAGTGCAGGTAG | bta-miR-652 | | AATGGCGCCACTAGGGTTGTG | |
| bta-miR-19a | TGTGCAAATCTATGCAAAACTGA | bta-miR-551b | | GGCGACCCATACTTGGTTTCAG | |
| bta-miR-20b | CAAAGTGCTCACAGTGCAGGTA | bta-miR-653 | | GTGTTGAAACAATCTCTGTTG | |
| bta-miR-19b | TGTGCAAATCCATGCAAAACTGA | bta-miR-562 | | AAAGCAGCTGTACCATTTAC | |
| bta-miR-21-3p | AACAGCAGTCGATGGGCTGTCT | bta-miR-654 | | TATGTCTGCTGACCATCACCTT | |
| bta-miR-200a | TAACACTGTCTGGTAACGATGTT | bta-miR-568 | | ATGTATAAATGTATACACAC | |
| bta-miR-21-5p | TAGCTTATCAGACTGATGTTGACT | bta-miR-655 | | ATAATACATGGTTAACCTCTCT | |
| bta-miR-200b | TAATACTGCCTGGTAATGATG | bta-miR-574 | | TGAGTGTGTGTGTGTGAGTGTGTG | |
| bta-miR-210 | ACTGTGCGTGTGACAGCGGCTGA | bta-miR-656 | | AATATTATACAGTCAACCTCT | |
| bta-miR-211 | TTCCCTTTGTCATCCTTTGCC | bta-miR-658 | | GGCGGAGGGAAGCGGGTCCGTTGGT | |
| bta-miR-22-5p | AGTTCTTCAGTGGCAAGCTTTA | bta-miR-758 | | TTTGTGACCTGGTCCACTAACC | |
| bta-miR-212 | ACCTTGGCTCTAGACTGCTTACT | bta-miR-660 | | TACCCATTGCATATCGGAGCTG | |
| bta-miR-221 | AGCTACATTGTCTGCTGGGTTT | bta-miR-759 | | GCAGACTGCAAACAATTTTGAC | |
| bta-miR-214 | ACAGCAGGCACAGACAGGCAGT | bta-miR-664a | | CAGGCTGGGGTGTGTGTGGATG | |
| bta-miR-222 | AGCTACATCTGGCTACTGGGT | bta-miR-760-3p | | CGGCTCTGGGTCTGTGGGGA | |
| bta-miR-215 | ATGACCTATGAATTGACAGACA | bta-miR-664b | | TATTCATTTATCTCCCAGCCTAC | |
| bta-miR-223 | TGTCAGTTTGTCAAATACCCCA | bta-miR-760-5p | | CCCCTCAGTCCACCAGAGCCCG | |
| bta-miR-216a | TAATCTCAGCTGGCAACTGTGA | bta-miR-665 | | ACCAGTAGGCCGAGGCCCCT | |
| bta-miR-224 | CAAGTCACTAGTGGTTCCGTTTA | bta-miR-761 | | GCAGCAGGGTGAAACTGACACA | |
| bta-miR-216b | AAATCTCTGCAGGCAAATGTGA | bta-miR-669 | | TGTGGGTGTGTGCATGTGCGTG | |
| bta-miR-23a | ATCACATTGCCAGGGATTTCCA | bta-miR-763 | | CCAGCTGGGAGGAACCAGTGGC | |
| bta-miR-217 | TACTGCATCAGGAACTGATTGGAT | bta-miR-670 | | TCCCTGAGTATATGTGGTGAA | |
| bta-miR-23b-3p | ATCACATTGCCAGGGATTACCAC | bta-miR-764 | | GGTGCTCACTCGTCCTTCT | |
| bta-miR-218 | TTGTGCTTGATCTAACCATGTG | bta-miR-671 | | AGGAAGCCCTGGAGGGGCTGGAG | |
| bta-miR-23b-5p | GGGTTCCTGGCATGCTGATTT | bta-miR-767 | | TGCACCATGGTTGTCTGAGCATG | |
| bta-miR-219 | AGAGTTGAGTCTGGACGTCCCG | bta-miR-677 | | CTCACTGATGAGCAGCTTCTGAC | |
| bta-miR-24 | GTGCCTACTGAGCTGATATCAGT | bta-miR-769 | | TGAGACCTCCGGGTTCTGAGCT | |
| bta-miR-219-3p | AGAATTGTGGCTGGACATCTG | bta-miR-7 | | TGGAAGACTAGTGATTTTGTTGTT | |
| bta-miR-24-3p | TGGCTCAGTTCAGCAGGAACAG | bta-miR-873 | | GCAGGAACTTGTGAGTCTCCT | |
| bta-miR-219-5p | TGATTGTCCAAACGCAATTCTT | bta-miR-708 | | AAGGAGCTTACAATCTAGCTGGG | |
| bta-miR-25 | CATTGCACTTGTCTCGGTCTGA | bta-miR-874 | | CTGCCCTGGCCCGAGGGACCGA | |
| bta-miR-22-3p | AAGCTGCCAGTTGAAGAACTG | bta-miR-744 | | TGCGGGGCTAGGGCTAACAGCA | |
| bta-miR-26a | TTCAAGTAATCCAGGATAGGCT | bta-miR-875 | | TATACCTCAGTTTTATCAGGTG | |
| bta-miR-26b | TTCAAGTAATTCAGGATAGGTT | bta-miR-876 | | TGGATTTCTTTGTGAATCACCA | |
| bta-miR-29d-3p | TAGCACCATTTGAAATCGATTA | bta-miR-98 | | TGAGGTAGTAAGTTGTATTGTT | |
| bta-miR-26c | AGCCTATCCTGGATTACTTGAA | bta-miR-877 | | GTAGAGGAGATGGCGCAGGG | |
| bta-miR-29d-5p | TGACCGATTTCTCCTGGTGTT | bta-miR-99a-3p | | CAAGCTCGCTTCTATGGGT | |
| bta-miR-27a-3p | TTCACAGTGGCTAAGTTCCG | bta-miR-885 | | TCCATTACACTACCCTGCCTCT | |
| bta-miR-29e | TAGCATCATTTGAAATCAGTGTTT | bta-miR-99a-5p | | AACCCGTAGATCCGATCTTGT | |
| bta-miR-27a-5p | AGGGCTTAGCTGCTTGTGAGCA | bta-miR-9-3p | | ATAAAGCTAGATAACCG | |
| bta-miR-301a | CAGTGCAATAGTATTGTCAAAGCAT | bta-miR-99b | | CACCCGTAGAACCGACCTTGCG | |
| bta-miR-27b | TTCACAGTGGCTAAGTTCTGC | bta-miR-9-5p | | TCTTTGGTTATCTAGCTGTATG | |
| bta-miR-301b | CAGTGCAATGATATTGTCAAAGCAT | bta-miR-1179 | | AAGCATTCTTTCATTGGTTGG | |
| bta-miR-28 | AAGGAGCTCACAGTCTATTGAG | bta-miR-92a | | TATTGCACTTGTCCCGGCCTGT | |
| bta-miR-302a | AAGTGCTTCCATGTTTTAGTGA | bta-miR-1185 | | AGAGGATACCCTTTGTATGTT | |
| bta-miR-296-3p | GAGGGTTGGGCGGAGGCTTTCC | bta-miR-92b | | TATTGCACTCGTCCCGGCCTCC | |
| bta-miR-302b | TAAGTGCTTCCATGTTTTAGTAG | bta-miR-1193 | | TAGGTCACCCGTTTGACTATC | |
| bta-miR-296-5p | GAGGGCCCCCCCCAATCCT | bta-miR-93 | | CAAAGTGCTGTTCGTGCAGGTA | |
| bta-miR-302c | TAAGTGCTTCCATGTTTCAGTGG | bta-miR-1197 | | TAGGACACATGGTCTACTTCT | |
| bta-miR-299 | TGGTTTACCGTCCCACATACAT | bta-miR-935 | | CCAGTTACCGCTTCCGCTACCGC | |
| bta-miR-302d | TAAGTGCTTCCATGTTTTAGT | bta-miR-122 | | TGGAGTGTGACAATGGTGTTTG | |
| bta-miR-29a | CTAGCACCATCTGAAATCGGTTA | bta-miR-940 | | AAGGCTGGGCCCCCGCTCCGC | |
| bta-miR-3064 | TTGCCACACTGCAACACCTTACA | bta-miR-1224 | | GTGAGGACTCGGGAGGTGGAG | |
| bta-miR-29b | TAGCACCATTTGAAATCAGTGTT | bta-miR-95 | | TTCAACGGGTATTTATTGAGCA | |
| bta-miR-30a-5p | TGTAAACATCCTCGACTGGAAGCT | bta-miR-1225-3p | | CCGAGCCCCTGTGCCGCCCCCAG | |
| bta-miR-29c | TAGCACCATTTGAAATCGGTTA | bta-miR-96 | | TTTGGCACTAGCACATTTTTGCT | |
| bta-miR-30b-3p | CTGGGAGGTGGATGTTTACTT | bta-miR-1246 | | AATGGATTTTTGGAGCAGG | |
| bta-miR-30b-5p | TGTAAACATCCTACACTCAGCT | bta-miR-1247-3p | | CGGGAACGTCGGGACTGGAGC | |
| bta-miR-328 | CTGGCCCTCTCTGCCCTTCCGT | bta-miR-1296 | | TTAGGGCCCTGGCTCCATCTCC | |
| bta-miR-30c | TGTAAACATCCTACACTCTCAGC | bta-miR-1247-5p | | ACCCGTCCCGTGCGTCCCCGGA | |
| bta-miR-329a | AACACACCTGGTTAACCTTTTT | bta-miR-1298 | | TTCATTCGGCTGTCCAGATGTA | |
| bta-miR-30d | TGTAAACATCCCCGACTGGAAGCT | bta-miR-1248 | | ACCTTCTTGTATAAGCACTGTGCTAAA | |
| bta-miR-329b | AGAGGTTTTCTGGGTTTCTGTTT | bta-miR-1301 | | TTGCAGCTGCCTAGGAGTGATTTC | |
| bta-miR-30e-5p | TGTAAACATCCTTGACTGGAAGCT | bta-miR-1249 | | ACGCCCTTCCCCCCCTTCTTCA | |
| bta-miR-330 | GCAAAGCACACGGCCTGCAGAGA | bta-miR-1306 | | CCACCTCCCCTGCAAACGTCC | |
| bta-miR-30f | TGTAAACACCCTACACTCTCAGCT | bta-miR-1260b | | ATCCCACCACTGCCACCA | |
| bta-miR-331-3p | GCCCCTGGGCCTATCCTAGAA | bta-miR-1307 | | ACTCGGCGTGGCGTCGGTCGTG | |
| bta-miR-31 | AGGCAAGATGCTGGCATAGCT | bta-miR-1271 | | CTTGGCACCTAGTAAGTACTCA | |
| bta-miR-331-5p | TCTAGGTATGGTCCCAGG | bta-miR-1343-3p | | CTCCTGGGGCCCGCACTCTC | |
| bta-miR-32 | TATTGCACATGACTAAGTTGCAT | bta-miR-1277 | | TACGTAGATATATATGTATTTT | |
| bta-miR-335 | TCAAGAGCAATAACGAAAAATGT | bta-miR-1343-5p | | TGGGGAGCGGCCCCCGGGCGGG | |
| bta-miR-320a | AAAAGCTGGGTTGAGAGGGCGA | bta-miR-1281 | | TCGCCTCCTCCTCTCCC | |
| bta-miR-338 | TCCAGCATCAGTGATTTTGTTGA | bta-miR-1388-3p | | ATCTCAGGTTTGTCAGCCCGCA | |
| bta-miR-320b | AGCTGGGTTGAGAGGGTGGT | bta-miR-1282 | | TCGTTTGCCTTTTTCTGCTT | |
| bta-miR-339a | TCCCTGTCCTCCAGGAGCTCAC | RNT43 snoRNA | | CTTATTGACGGGCGGACAGAAAC | |
| bta-miR-323 | GCACATTACACGGTCGACCTCT | bta-miR-1284 | | TCTGCACAGACCCTGGCTTTTC | |
| bta-miR-339b | TCCCTGTCCTCCAGGAGCTC | Hm/Ms/Rt T1 snRNA | | CGACTGCATAATTTGTGGTAGTGG | |
| bta-miR-324 | CGCATCCCCTAGGGCATTGGTGT | bta-miR-1287 | | TGCTGGATCAGTGGTTTGAGTC | |
| bta-miR-33a | GTGCATTGTAGTTGCATTGCA | bta-miR-99b | | CACCCGTAGAACCGACCTTGCG | |
| bta-miR-326 | CCTCTGGGCCCTTCCTCCAG | bta-miR-1291 | | TGGCCCTGACTGAAGACCTGCAGT | |
| bta-miR-33b | GTGCATTGCTGTTGCATTGC |  |  | |  |
